# Supplementary material for: A pseudotyped adenovirus serotype 5 vector with serotype 49 fiber knob is an effective vector for vaccine and gene therapy applications
Source: Mol Ther Methods Clin Dev. 2024 Jul 30;32(3):101308. doi: 10.1016/j.omtm.2024.101308 (PMC11357811; doi:10.1016/j.omtm.2024.101308)
Supplement: Document S1. Figures S1–S6, Tables S1, and S2 [file mmc1.pdf]

OMTM, Volume *32*

Supplemental information

A pseudotyped adenovirus serotype 5 vector with serotype 49 fiber knob is an effective vector for vaccine and gene therapy applications

Carly M. Bliss, Sarah L. Hulin-Curtis, Marta Williams, Mahulena Marušková, James A. Davies, Evelina Statkute, Alexander T. Baker, Louise Stack, Lucas Kerstetter, Lauren E. Kerr-Jones, Kate F. Milward, Gabrielle Russell, Sarah J. George, Luned M. Badder, Richard J. Stanton, Lynda Coughlan, Ian R. Humphreys, and Alan L. Parker

Table S1 – Flow cytometry antibody panel used in Figure 3

Marker	Staining Cocktail	Fluorochrome	Antibody Clone	Dilution	Stock Concentration	Source
**CD3**	Surface	Pacific Blue	17A2	1:100	0.5mg/mL	Biolegend
**CD4**	Surface	BV510	RM4-5	1:150	0.2mg/mL	Biolegend
**CD8**	Surface	AlexaFluor700	53-6.7	1:50	0.2mg/mL	eBioscience
**LIVE/ DEAD**	Surface	Near Infrared	N/A	1:1000	N/A	ThermoFisher
**IL-2**	Intracellular	PE-Cy7	JES6-5H4	1:250	0.2mg/mL	eBioscience
**IFN-γ**	Intracellular	eFluor660	XMG1.2	1:500	0.2mg/mL	eBioscience
**TNFα**	Intracellular	AlexaFluor488	MP6-XT22	1:2000	0.5mg/mL	eBioscience
**CD40L**	Intracellular	PerCP eFluor710	MR1	1:150	0.2mg/mL	ThermoFisher

Table S2 – Flow cytometry antibody panel used in Figure 5

Marker	Staining Cocktail	Fluorochrome	Antibody Clone	Dilution	Stock Concentration	Source
**CD4**	Surface	APC	GK1.5	1:100	0.2mg/mL	Biolegend
**CD8**	Surface	PE-Cy7	53-6.7	1:50	0.2mg/mL	Biolegend
**LIVE/ DEAD**	Surface	Zombie Aqua	N/A	1:500	N/A	Biolegend
**IFN-γ**	Intracellular	FITC	XMG1.2	1:100	0.5mg/mL	Biolegend
**TNFα**	Intracellular	PE	MP6-XT22	1:100	0.2mg/mL	Biolegend

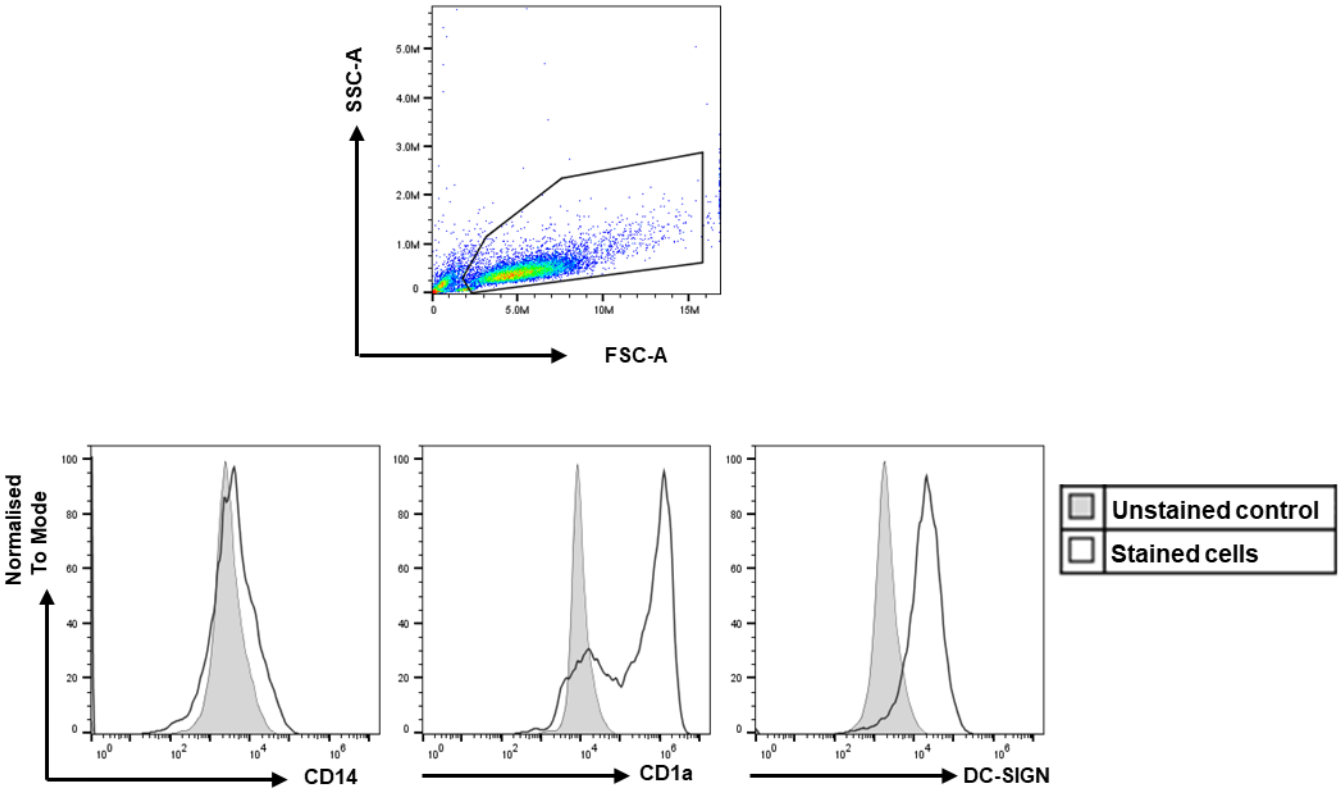

Figure S1 – Validation of human monocyte-derived dendritic cells using surface marker expression

Following *in vitro* differentiation from CD14$^+$ enriched PBMC, dendritic cell phenotype was confirmed by flow cytometry using CD14$^-$ CD1a$^+$ DC-SIGN$^+$ validation.

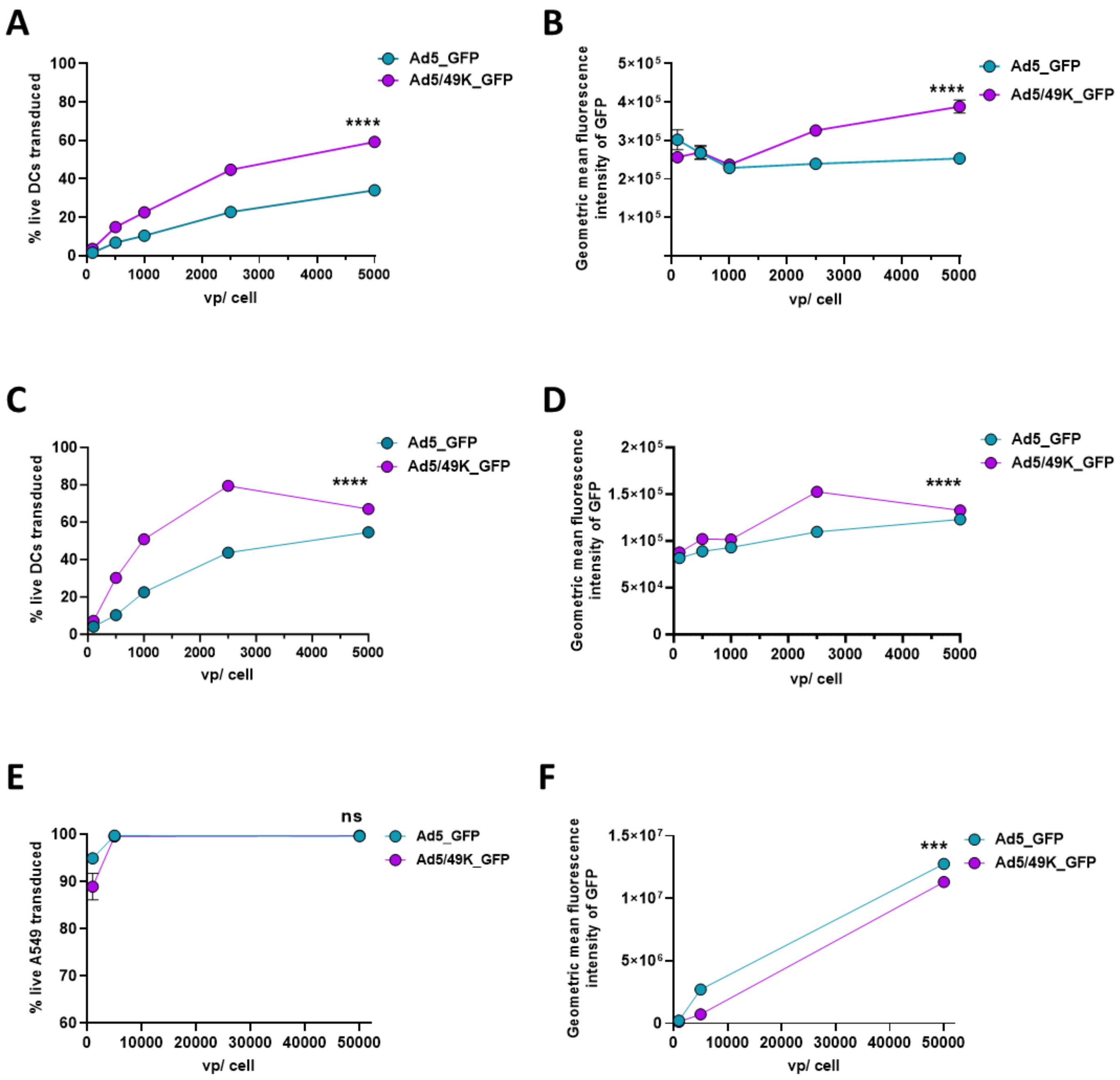

Figure S2 – Area-under-curve analyses of dendritic cell transduction

A-F) Dose response data used to calculate area-under-curve (AUC) of transduction assay data presented in corresponding ***Figure 2A-F***. AUC mean with standard error of the mean (SEM) compared by unpaired T test. Mean and SEM displayed. **** p<0.0001, *** p<0.001, ns = no significant difference.

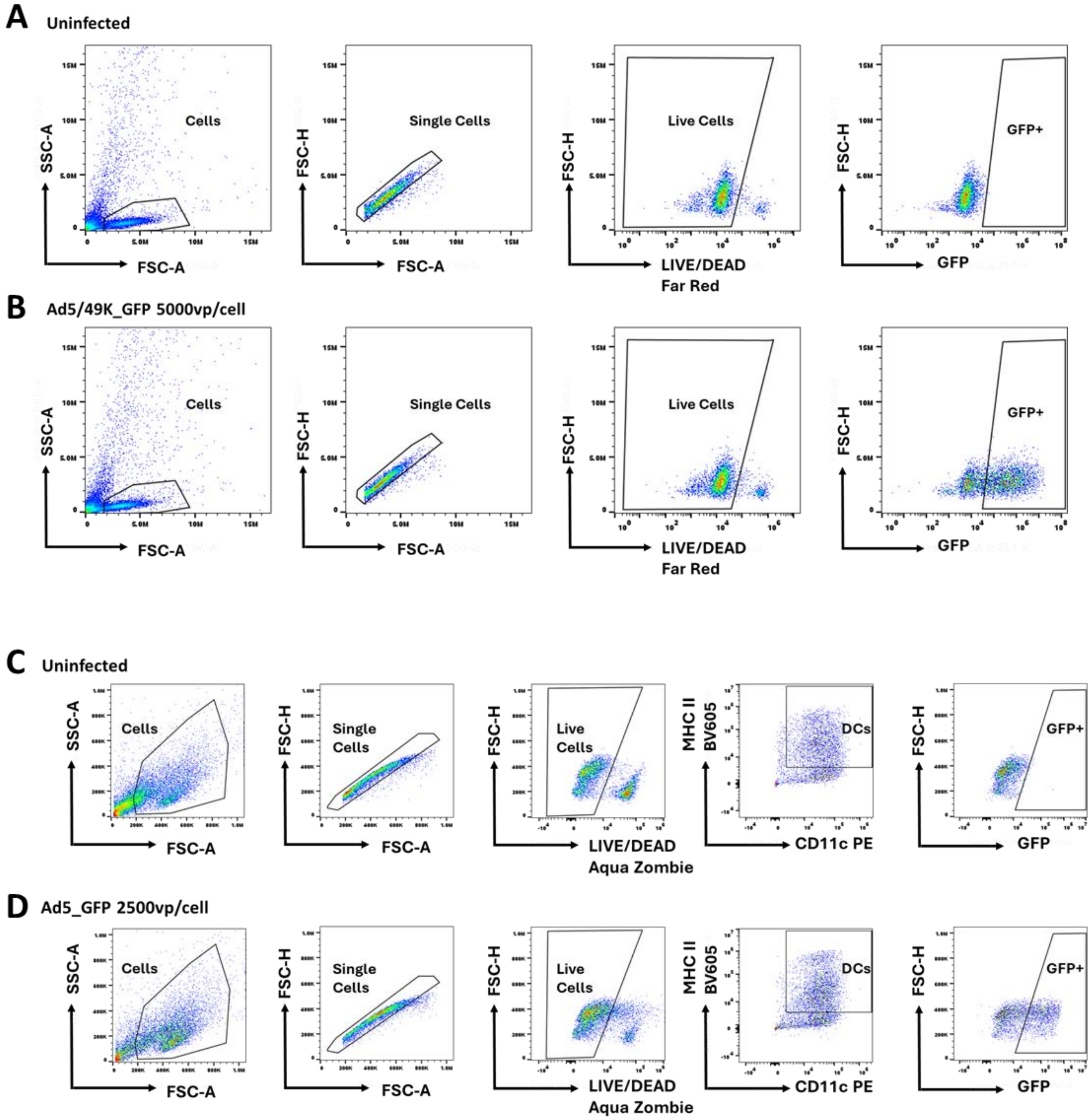

Figure S3 – Human and murine dendritic cell gating strategy for *in vitro* virus transduction assays using GFP-expressing vectors.

Representative gating strategy using Far Red dead cell staining and GFP $^{+}$ gating for **A)** Uninfected monocyte-derived human DCs and **B)** Monocyte-derived human DCs infected with Ad5/49K_GFP at 5,000vp/cell. Representative gating strategy using Aqua Zombie dead cell staining and GFP^{+} gating for **C)** Uninfected bone marrow-derived murine DCs and **D)** Bone-marrow-derived murine DCs infected with Ad5_GFP at 2,500vp/cell.

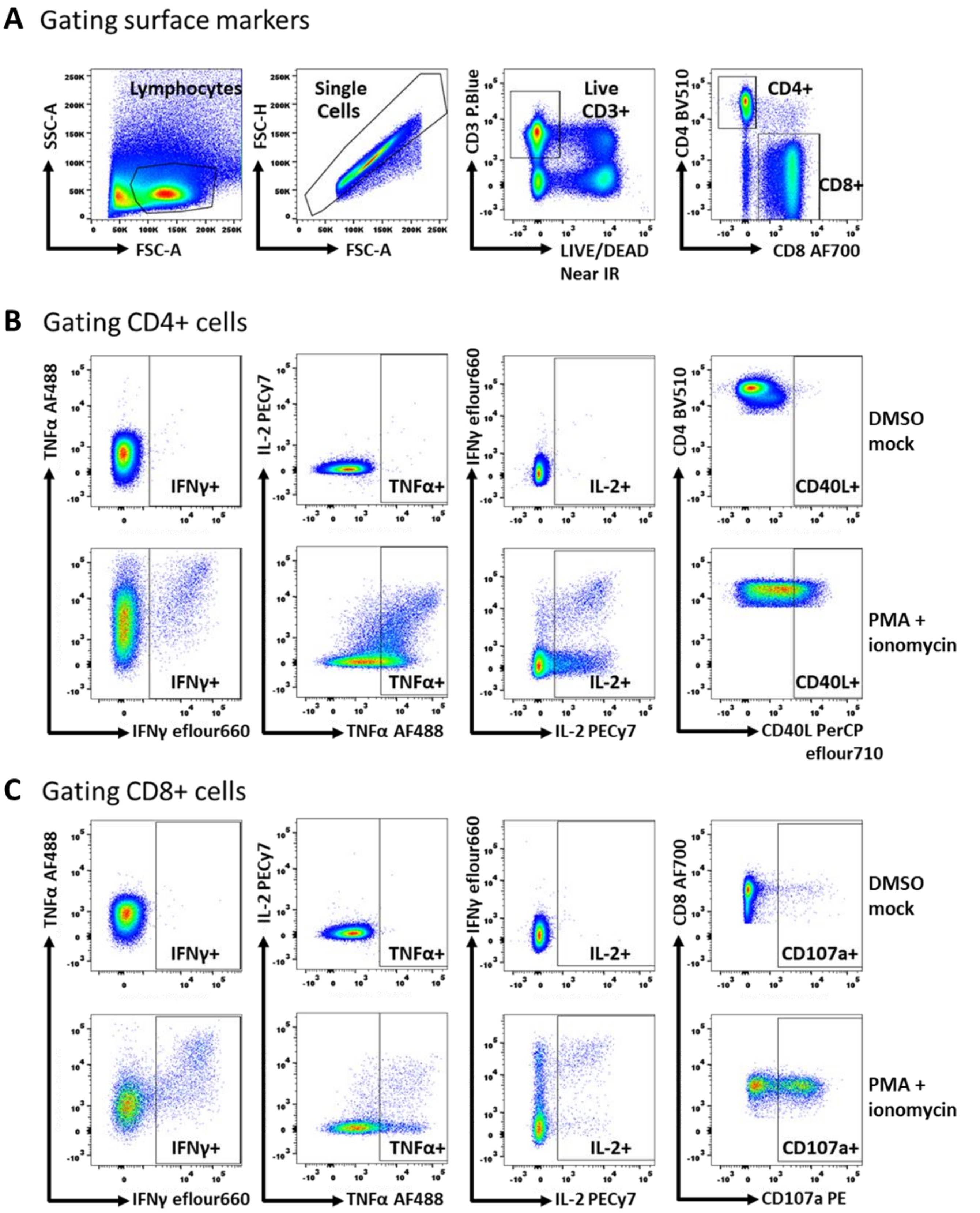

Figure S4 – Gating strategy for flow cytometry with intracellular cytokine staining for vectored vaccines against GFP.

Representative gating strategy for identification of **A)** live $CD4^+$ and $CD8^+$ T cells, with downstream cytokine/CD40L/CD107a responses shown for **B)** $CD4^+$ and **C)** $CD8^+$ populations stimulated with DMSO (-ve control) and PMA+ionomycin (+ve control).

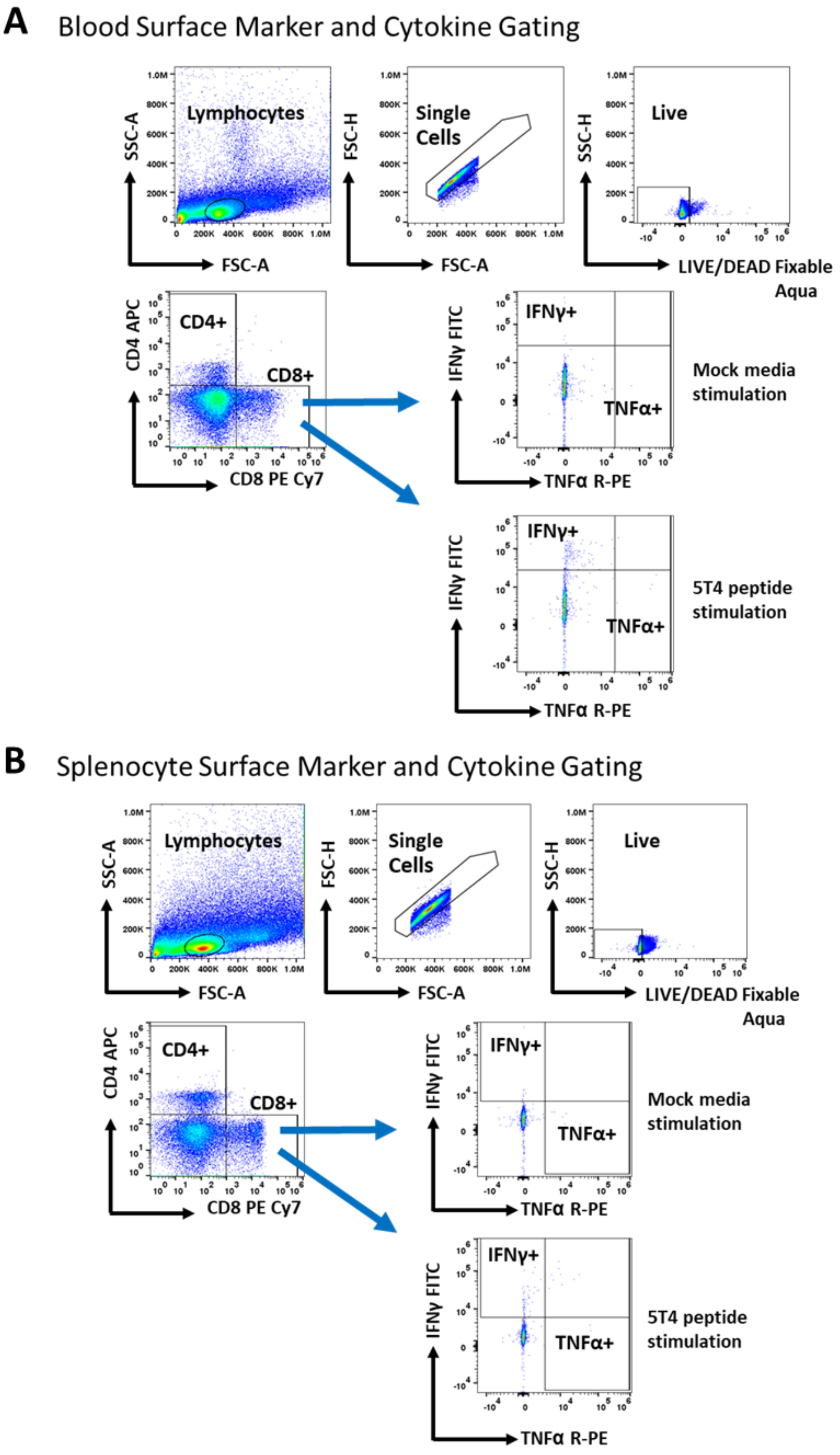

Figure S5 – Gating strategy for flow cytometry with intracellular cytokine staining for vectored vaccines against 5T4.

Representative gating strategy for **A)** PBMC and **B)** splenocyte flow cytometry identification of $CD8^{+}$ T cells with intracellular cytokine staining for media mock (-ve control) and 5T4 peptide stimulated cells.

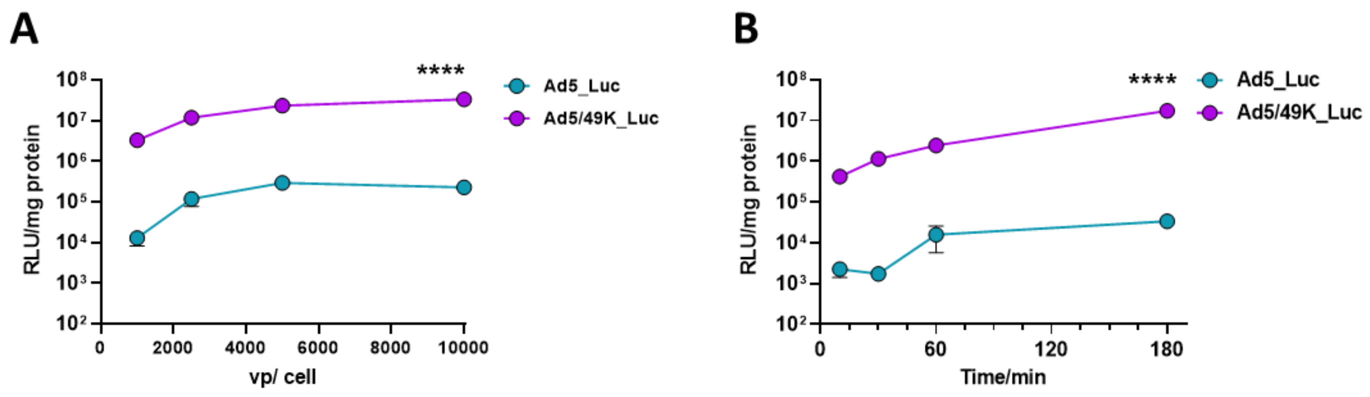

Figure S6 – Area-under-curve analyses of primary human vascular smooth muscle cell transduction.

A-B) Dose response data used to calculate area-under-curve (AUC) of transduction assay data presented in corresponding ***Figure 6A-B***. AUC mean with standard error of the mean (SEM) compared by unpaired T test. Mean and SEM displayed. **** $p<0.0001$.
