# Supplementary material for: A pseudotyped adenovirus serotype 5 vector with serotype 49 fiber knob is an effective vector for vaccine and gene therapy applications
Source: Mol Ther Methods Clin Dev. 2024 Jul 30;32(3):101308. doi: 10.1016/j.omtm.2024.101308 (PMC11357811; doi:10.1016/j.omtm.2024.101308)
Supplement: Document S2. Article plus supplemental information [file mmc2.pdf]

Molecular Therapy
Methods & Clinical Development
Original Article

A pseudotyped adenovirus serotype 5 vector with serotype 49 fiber knob is an effective vector for vaccine and gene therapy applications

Carly M. Bliss,[1,2] Sarah L. Hulin-Curtis,[1] Marta Williams,[3] Mahulena Marušková,[1] James A. Davies,[1] Evelina Statkute,[3] Alexander T. Baker,[1] Louise Stack,[1] Lucas Kerstetter,[4] Lauren E. Kerr-Jones,[3] Kate F. Milward,[1] Gabrielle Russell,[4] Sarah J. George,[5] Luned M. Badder,[1] Richard J. Stanton,[2,3] Lynda Coughlan,[4,6] Ian R. Humphreys,[2,3] and Alan L. Parker[1,2]

[1]Division of Cancer and Genetics, Cardiff University School of Medicine, Heath Park, Cardiff CF14 4XN, UK; [2]Systems Immunity University Research Institute, Cardiff University School of Medicine, Heath Park, Cardiff CF14 4XN, UK; [3]Division of Infection and Immunity, Cardiff University School of Medicine, Heath Park, Cardiff CF14 4XN, UK; [4]University of Maryland School of Medicine, Department of Microbiology and Immunology, Baltimore, MD 21201, USA; [5]Bristol Medical School, Translational Health Sciences, University of Bristol, Bristol BS2 8HW, UK; [6]University of Maryland School of Medicine, Center for Vaccine Development and Global Health, Baltimore, MD 21201, USA

Adenoviruses (Ads) have demonstrated significant success as replication-deficient (RD) viral vectored vaccines, as well as broad potential across gene therapy and cancer therapy. Ad vectors transduce human cells via direct interactions between the viral fiber knob and cell surface receptors, with secondary cellular integrin interactions. Ad receptor usage is diverse across the extensive phylogeny. Commonly studied human Ad serotype 5 (Ad5), and chimpanzee Ad-derived vector "ChAdOx1" in licensed ChAdOx1 nCoV-19 vaccine, both form primary interactions with the coxsackie and adenovirus receptor (CAR), which is expressed on human epithelial cells and erythrocytes. CAR usage is suboptimal for targeted gene delivery to cells with low/negative CAR expression, including human dendritic cells (DCs) and vascular smooth muscle cells (VSMCs). We evaluated the performance of an RD Ad5 vector pseudotyped with the fiber knob of human Ad serotype 49, termed Ad5/49K vector. Ad5/49K demonstrated superior transduction of murine and human DCs over Ad5, which translated into significantly increased T cell immunogenicity when evaluated in a mouse cancer vaccine model using 5T4 tumor-associated antigen. Additionally, Ad5/49K exhibited enhanced transduction of primary human VSMCs. These data highlight the potential of Ad5/49K vector for both vascular gene therapy applications and as a potent vaccine vector.

INTRODUCTION

Adenoviruses (Ads) are a family of viruses that generally cause mild, transient disease in immunocompetent individuals, including conjunctivitis and infections of the respiratory, urinary, and gastrointestinal tracts. Over 100 human Ad genotypes segregate into more than 50 distinct serotypes that are phylogenetically and phenotypically categorized into seven species, termed A–G.[1,2] The safety profile of Ad delivery can be enhanced through well-characterized deletions in early viral genes, resulting in replication-deficient (RD) Ad vectors capable of efficient transgene delivery.[3] These vectors can be rapidly amplified to high, clinically applicable titers, with their genetic manipulation extensively studied for vaccine, gene therapy, and cancer applications. Species C human Ad serotype 5 (Ad5) has historically been the most clinically evaluated Ad; however, this represents a fraction of Ad serotypes available, which exhibit diverse patterns of receptor usage and translational potential. This highlights the need to explore alternative Ad serotypes and pseudotypes for the multitude of applications where Ads have already shown promise.

RD Ad vectored vaccines exhibit excellent clinical immunogenicity with multiple human and non-human Ad serotypes having demonstrated induction of potent adaptive immune response consisting of both antigen-specific T cells and antibodies.[4–7] Such balanced and broad immunity is a highly desirable characteristic for Ad-based infectious-disease vaccines under clinical development.[7–11] This is highlighted by their recent licensure as COVID-19 vaccines using a vector derived from human species D Ad serotype 26 (Ad26.COV2-S/Jcovden) and using the species E chimpanzee Ad-derived "ChAdOx1" vector (*ChAdOx1 nCoV-19*/Vaxzevria AZD1222), both delivered via the intramuscular (i.m.) route.[12–16] Additionally, a ChAd36-based COVID-19 vaccine (iNCOVACC) has local authorization for intranasal administration in India.[17] Non-Ad5-based vectors often exhibit reduced immunogenicity when compared to Ad5 in pre-clinical and clinical settings,[18–20] suggesting Ad5 is a "gold standard" immunogenic vaccine vector. Despite some clinical vaccine studies citing limitations relating to

Received 20 January 2024; accepted 26 July 2024;
https://doi.org/10.1016/j.omtm.2024.101308.

Correspondence: Alan L. Parker, Division of Cancer and Genetics, Cardiff University School of Medicine, Heath Park, Cardiff CF14 4XN, UK.
E-mail: parkeral@cardiff.ac.uk

© 2024 The Author(s). Published by Elsevier Inc. on behalf of The American Society of Gene and Cell Therapy. This is an open access article under the CC BY license (http://creativecommons.org/licenses/by/4.0/).

pre-existing anti-Ad5 immunity,[21,22] Ad5-based vectors have also been authorized as stand-alone COVID-19 vaccines and as part of a two-dose vaccine regimen paired with Ad26 vector.[23,24] In addition to COVID-19 vaccines, Ad vector research and development extends to many other infectious diseases and to therapeutic cancer vaccines, due to their potent induction of cytotoxic CD8$^+$ T cell immunity.[25,26]

The Ad capsid consists of three major structural proteins: the hexon protein, the fiber with a shaft and knob domain, and the penton base from which the fiber protrudes. Classical Ad5 infection is initiated via a primary interaction between the fiber knob and the cellular coxsackie and adenovirus receptor (CAR),[27] which is expressed on human epithelial cells and erythrocytes.[28,29] CAR is a high-affinity receptor across Ad species, including for ChAdOx1.[30] This primary receptor interaction is followed by secondary interactions of the penton base with cellular integrins, which triggers cell entry.[31] Different Ad serotypes can have distinct receptor tropisms, with differentially expressed receptors already described for several Ad serotypes.[32–36] Switching the fiber-knob domain with that of a different Ad serotype can therefore alter cellular tropism.

Human Ad serotype 49 (Ad49) is a species D Ad with low global seroprevalence.[37,38] It has previously been evaluated as a pre-clinical vaccine vector exhibiting low cross-reactivity with Ad5 immunity; however, its native vaccine immunogenicity in naive (non-Ad5 pre-exposed) animals was significantly lower than for Ad5.[38] We previously developed a novel chimeric Ad, termed Ad5/49K, consisting of an RD Ad5 vector pseudotyped with the fiber-knob domain of Ad49, generated to study receptor usage of the Ad49 fiber knob. It was deemed promiscuous, with CAR usage reported as unlikely following highly efficient Ad5/49K vector transduction of CHO-K1 (CAR-negative) cells.[34] Exploiting alternative receptor usage to CAR through altering the fiber-knob domain represents a strategy for targeted and more immunogenic vector applications. The Ad5/49K vector could therefore provide an optimal "middle ground" in terms of retaining the potent immunogenicity of Ad5 as a vaccine vector while harnessing the differential receptor tropisms of Ad49 to transduce different cell types for robust immune priming or cellular transduction.

Cellular immune priming is underpinned by antigen presentation to naive T cells via professional antigen-presenting cells (APCs), such as dendritic cells (DCs). In the context of vaccination, DCs can present antigen to T cells and provide sufficient co-stimulation to prime an immunogenic, adaptive T cell response. CD8$^+$ T cells are primed via major histoompatibility complex (MHC) class I-mediated antigen presentation from APCs, via direct presentation of endogenous peptides and via cross-presentation of exogenous peptides. Conversely, CD4$^+$ T cells are typically primed via MHC class II-mediated antigen presentation of exogenous peptides via APCs. *Ex vivo* priming of human monocyte-derived DCs is successfully used in sipuleucel-T therapy, where autologous DCs are stimulated with a fusion protein and re-infused into patients as a U.S. Food and Drug Administration- (FDA) approved cellular immunotherapy for prostate cancer.[39] *In vivo* targeting of antigen to DCs has also demonstrated enhanced immunogenicity pre-clinically,[40] with such approaches as ligand-, antibody-, and vector-based DC targeting.[41] Human Langerhans cells and dermal DCs express CAR; however, the receptor is absent on human plasmacytoid DCs and monocyte-derived DCs.[42] CAR-utilizing Ads, such as Ad5 and ChAdOx1, must utilize alternative pathways to mediate entry into CAR-negative DCs to deliver their encoded antigen directly,[42–44] or rely on indirect cross-presentation of the Ad-encoded antigen to T cells. Ads with non-CAR receptor usage may transduce DCs more efficiently, resulting in increased direct antigen presentation to T cells, with potential for enhanced immune priming. We evaluated herein using i.m. and intravenous (i.v.) vaccination models with Ad5/49K vector in mice.

Finally, Ad49 exhibits tropism toward cells of the human vasculature, with significantly enhanced transduction in comparison to Ad5,[45] highlighting the potential vascular gene transfer applications of the Ad5/49K vector. In patients with coronary artery disease, autologous human saphenous vein (HSV) grafts are frequently used; however, by 10 years post surgery, patency rates for HSV grafts are approximately 50% and only half of those that remain non-occluded have no signs of atherosclerosis.[46] Activation of vascular smooth muscle cells (VSMCs) within the HSV grafts occurs as a result of the increased shear stress and pressure within the arterial circulation and results in enhanced VSMC proliferation and secretion of extracellular matrix proteins. Excessive VSMC activation leads to thickening of the intima of the graft and subsequent superimposed atherosclerosis and vein graft failure. Gene therapy represents an excellent opportunity for therapeutic intervention to prevent changes associated with vascular remodeling, with a window of opportunity for *ex vivo* gene therapy application afforded while the graft is outside the body between harvesting and implantation. The success of Ad-based cardiovascular gene therapy to date has been limited by a lack of Ad5 tropism to CAR-negative VSMCs,[47] resulting in high-titer Ad requirements. Therefore, alongside potential vaccine applications, we also evaluated the Ad5/49K vector in the context of vascular gene therapy.

RESULTS

Generation of Ad5 and Ad5/49K vectors expressing green fluorescent protein, 5T4 tumor-associated antigen, and luciferase

We previously reported the generation of a chimeric RD Ad5 vector with the fiber knob from Ad49 (Ad5/49K; Figure 1A) to study receptor interactions.[34] RD Ad5 and RD Ad5/49K vectors were generated to express green fluorescent protein (GFP), 5T4 oncofetal antigen (5T4, also known as trophoblast glycoprotein), or luciferase (Luc): Ad5_GFP, Ad5/49K_GFP, Ad5_5T4, Ad5/49K_5T4, Ad5_Luc, and Ad5/49K_Luc. All viral vectors were quantified by microBCA assay to determine virus particles (vp)/mL titer, as previously described where 1 μg of protein = 4×10^9 vp (Table 1).[47,48] The fiber regions of representative purified viruses were sequenced to confirm the successful incorporation of the chimeric fiber. The translated amino acid sequence of the fiber shaft remains identical between the Ad5 and Ad5/49K vectors and differs only after the shaft-knob hinge region identified by amino acids T-L-W (Figure 1B). Nanoparticle tracking analysis verified the virus

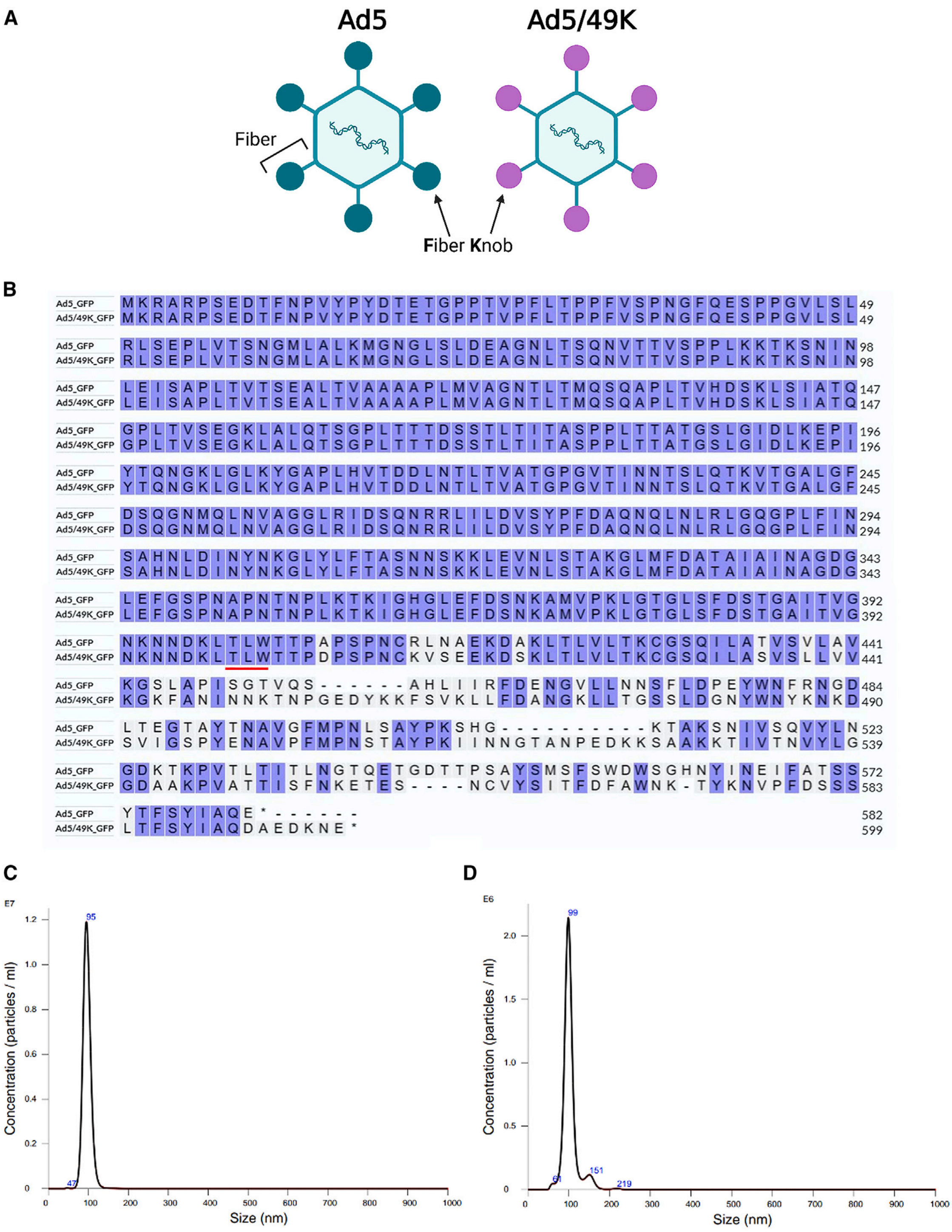

Figure 1. Generation of Ad5 and Ad5/49K vectors

(A) Recombineering technology was used to generate a chimeric vector with the Ad49 fiber-knob domain pseudotyped onto Ad5 vector. (B) Representative sequencing of Ad5_GFP and Ad5/49K_GFP confirms the fiber-knob swap, as depicted by the translated amino acid sequence. Fiber shaft:knob hinge region underlined in red. (C) Ad5_GFP and (D) Ad5/49K_GFP nanoparticle size distribution of viruses measured in liquid suspension by NanoSight nanoparticle analyzer.

Table 1. Titers of RD Ad5 and Ad5/49K vectors

Vector	Titer (vp/mL)	Experiment
Ad5_GFP #1<br>Ad5_GFP #2	5.60×10^{12}<br>3.69×10^{11}	Figures 3 and 4,<br>Figures 1B, 1C, and 2A–2F
Ad5/49K_GFP #1<br>Ad5/49K_GFP #2	1.33×10^{12}<br>2.74×10^{11}	Figures 3 and 4,<br>Figures 1B, 1D, and 2A–2F
Ad5_5T4	4.41×10^{12}	Figure 5
Ad5/49K_5T4	1.6×10^{12}	Figure 5
Ad5_Luc	2.9×10^{12}	Figure 6
Ad5/49K_Luc	1.4×10^{12}	Figure 6

Ad5 and Ad5/49K vectors with green fluorescent protein (GFP), 5T4 oncofetal antigen (5T4), and luciferase (Luc) transgenes. Vector name with batch number, vp/mL titer by microBCA assay and experiment number displayed. vp, virus particles.

particle size distribution of Ad5_GFP and Ad5/49_GFP. A dominant peak of 95 nm was measured for Ad5_GFP (Figure 1C) and 99 nm for Ad5/49K_GFP (Figure 1D), which are within the expected range of intact Ad virions.

Ad5/49K vector exhibits enhanced transduction of primary human and murine DCs

Vectors that efficiently transduce and deliver encoded antigen to DCs could prime adaptive immune responses in a superior manner, making them more attractive as both prophylactic and therapeutic vaccine vectors. Ad5/49K_GFP was evaluated for transduction efficiency of human and murine DCs and compared with the equivalent parental Ad5_GFP. Human monocyte-derived DCs (hMo-DCs) were generated from an apheresis cone and validated by flow cytometry (Figure S1). In hMo-DCs, GFP transduction was statistically higher (Figure 2A, area under the curve [AUC] statistical analyses in Figure S2A), with statistically brighter fluorescence intensity of GFP signal suggesting a larger number of GFP molecules expressed on a per-cell basis using Ad5/49K compared to Ad5 (Figure 2B, AUC statistical analyses in Figure S2B). Equivalent transduction experiments were performed in murine CD11c^{+}MHCII^{+} bone marrow-derived DCs (mBM-DCs) and demonstrated similar results, with Ad5/49K_GFP exhibiting statistically higher transduction of mBM-DCs compared to Ad5_GFP (Figure 2C, AUC statistical analyses in Figure S2C), with more GFP expressed on a per-cell basis, as indicated by statistically higher fluorescence intensity of GFP signal per cell (Figure 2D, AUC statistical analyses in Figure S2D). Of note, almost 80% of mBM-DCs were transduced with 2,500 vp/cell of Ad5/49K_GFP, compared to just 44% with 2,500 vp/cell of Ad5_GFP.

In contrast to increased transduction of Ad5/49K over Ad5 in DCs, comparable transduction was observed between Ad5_GFP and Ad5/49K_GFP in an irrelevant human lung epithelial cell line (A549) (Figure 2E, AUC statistical analyses in Figure S2E). Transduction was measured using a high vp/cell range (1,000–50,000 vp/cell), which limited the scope to detect differences; however, statistically higher fluorescence intensity of GFP signal from Ad5_GFP over Ad5/49K_GFP indicated superior transfection in terms of GFP expression per A549 cell (Figure 2F, AUC statistical analyses in S2F). These data therefore suggest a degree of specificity to the enhanced Ad5/49K transduction measured in DCs.

Delivery of Ad5/49K as an i.m. vaccine induces comparable antigen-specific CD8^{+} T cell and antibody responses to Ad5

Prophylactic vaccines against infectious diseases are predominantly delivered via the i.m. route. Ad5/49K was therefore evaluated in a mouse i.m. vaccination model to assess induction of antigen-specific T cells against model antigen, GFP, 3 weeks post vaccination. GFP-specific CD4^{+} T cell responses in the spleen following Ad5_GFP and Ad5/49K_GFP vaccination showed the development of a T helper 1 (Th1) phenotype, as assessed by intracellular cytokine staining (ICS) following GFP peptide stimulation (Figures 3A–3C). Moreover, both vectors induced CD4^{+} T cell activation, as measured by CD40L expression (Figure 3D). No significant differences were measured between the frequencies of GFP-specific cytokine-producing or activated CD4^{+} T cells induced by Ad5_GFP and Ad5/49K_GFP.

T cell polyfunctionality is defined as the ability of a single T cell to carry out multiple functions, such as simultaneous secretion of cytokines, chemokines, or cytotoxic granules, as opposed to singular functions, termed monofunctionality. When evaluating the phenotypes of individual GFP-specific CD4^{+} T cells, both Ad5_GFP and Ad5/49K_GFP induced a dominant highly polyfunctional profile, with the major GFP-specific CD4^{+} T cell population consisting of interferon (IFN)-γ, interleukin (IL)-2, and tumor necrosis factor (TNF)α production, in addition to expression of CD40L in response to GFP peptide stimulation (Figure 3I). Of note, Ad5_GFP induced slightly elevated frequencies of monofunctional IFN-γ-producing CD4^{+} T cells compared with Ad5/49K_GFP, although this was not statistically significant.

The GFP-specific CD8^{+} T cell response was an order of magnitude greater than that of CD4^{+} T cells and was comparable across the Ad5_GFP- and Ad5/49K_GFP-vaccinated mice, with robust IFN-γ, IL-2, and TNFα responses measured in response to GFP peptide stimulation (Figures 3E–3G), in addition to a high frequency of CD8^{+} T cells expressing CD107a, a marker of cytotoxic degranulation (Figure 3H). This cytotoxic CD8^{+} T cell response was dominated by highly polyfunctional populations of cells capable of cytotoxic degranulation in addition to IFN-γ and TNFα production, alongside other polyfunctional phenotypes. The polyfunctional CD8^{+} T cell profile was tightly mirrored across the GFP-specific populations induced by Ad5_GFP and Ad5/49K_GFP (Figure 3J).

Serum samples taken at the same 3-week time point following i.m. vaccination with Ad5_GFP and Ad5/49K_GFP were evaluated for total immunoglobulin (Ig) G against recombinant GFP protein using ELISA with 3-fold dilution series from 1:100 serum (Figure 4A). Antibody endpoint titer above baseline was calculated, with baseline defined as mean plus 3 SD of naive mouse serum. No statistical difference in anti-GFP IgG endpoint titer was measured following Ad5_GFP and Ad5/49K_GFP vaccination, with both vectors inducing a robust anti-GFP IgG response after a single i.m. dose of vaccine (Figure 4B).

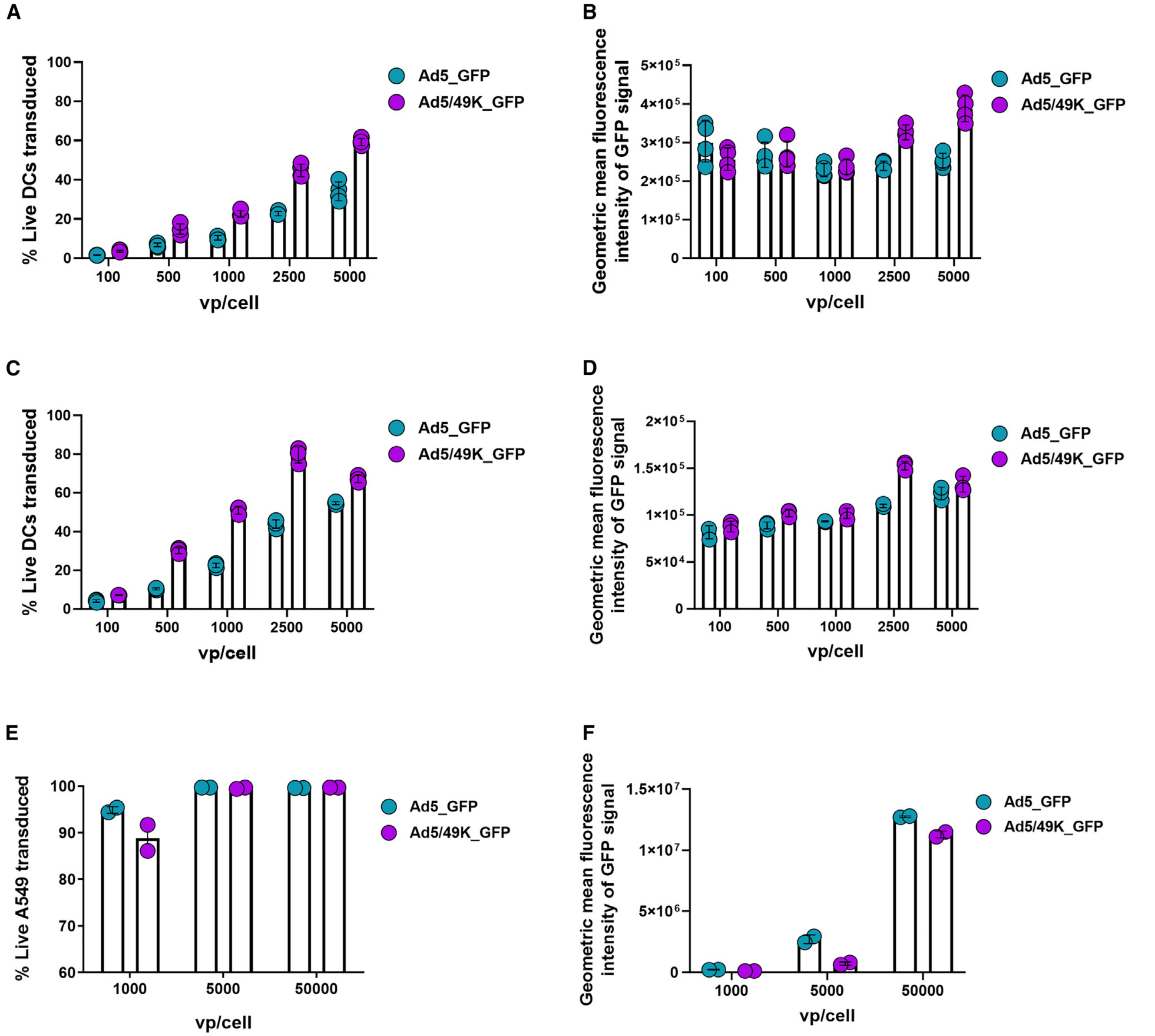

Figure 2. Ad5/49K vector exhibits enhanced transduction of primary human and murine DCs

(A) Percentage of live hMo-DCs transduced using 100–5,000 vp/cell of Ad5_GFP and Ad5/49K_GFP. $n = 4$/condition. (B) Geometric mean fluorescence intensity of GFP signal of GFP^+ cells identified in (A). (C) Percentage of live mBM-DCs transduced using 100–5,000 vp/cell of Ad5_GFP and Ad5/49K_GFP. $n = 3$/condition. (D) Geometric mean fluorescence intensity of GFP signal of GFP^+ cells identified in (C). (E) Percentage of live A549 cells 1,000–50,000 vp/cell of Ad5_GFP and Ad5/49K_GFP. $n = 2$/condition. (F) Geometric mean fluorescence intensity of GFP signal of GFP^+ cells identified in (E). Geometric mean with geometric SD displayed. Statistical analyses using AUC outlined in Figure S2.

Using the same serum samples from GFP-vaccinated mice, total IgG against Ad5 vector was measured using a whole inactivated Ad5 virus ELISA with 3-fold dilution series from 1:100 serum (Figure 4C), and the endpoint titer above baseline was calculated (Figure 4D). Anti-Ad5 endpoint titers were comparable between the Ad5_GFP- and Ad5/49K_GFP-vaccinated mice. Notably, anti-Ad5 IgG titers were 10- to 100-fold lower than the anti-GFP IgG titers.

Delivery of Ad5/49K as an i.v. cancer vaccine induces enhanced antigen-specific $CD8^+$ T cell immunity

5T4 is an oncofetal antigen expressed by many cancers yet is rarely expressed by normal adult tissue.[49] As a tumor-associated antigen (TAA), 5T4 is the target of several immunotherapeutic approaches, antibody-drug conjugates, chimeric antigen receptor T cell therapies, and cancer vaccines.[49] Robust induction and maintenance of

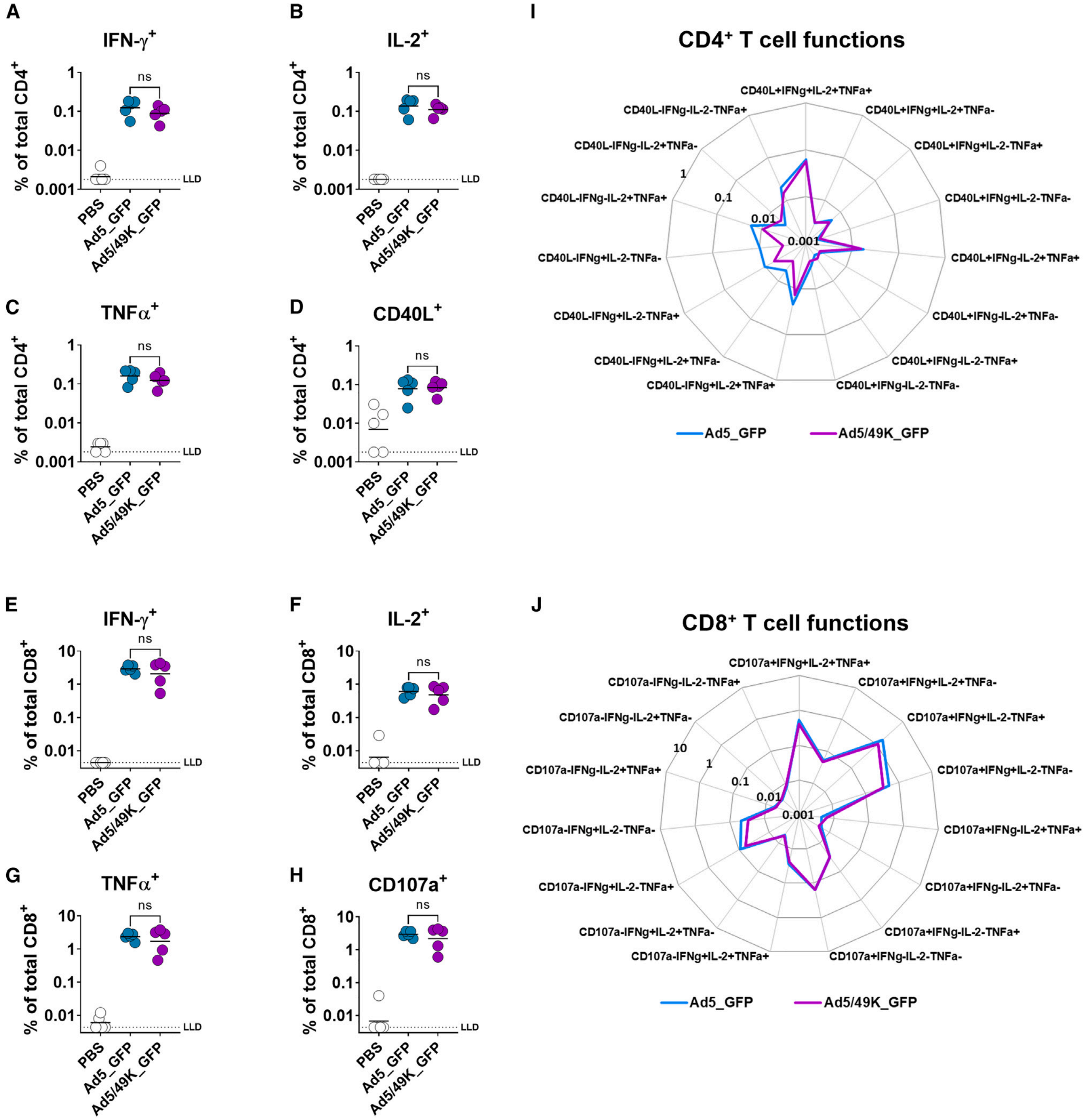

Figure 3. Ad5/49K vector induces comparable T cell immunogenicity and polyfunctionality to highly immunogenic Ad5 vector when administered as an i.m. vaccine to mice

Flow cytometry with ICS for GFP-specific $CD4^+$ (A) IFN-γ^+, (B) IL-2^+, (C) TNFα^+, and (D) $CD40L^+$ T cells, and GFP-specific $CD8^+$ (E) IFN-γ^+, (F) IL-2^+, (G) TNFα^+, and (H) $CD107a^+$ T cells following overlapping GFP peptide stimulation at day 21 following i.m. vaccination with PBS, 1×10^9 vp Ad5_GFP or 1×10^9 vp Ad5/49K_GFP. Line displayed at geometric mean based on frequencies of cytokine positive cells as a proportion of the total parent $CD4^+$ or $CD8^+$ T cell population. Kruskal-Wallis test comparing Ad5_GFP to Ad5/49K_GFP only. ns, no significant difference. LLD, lower limit of detection. Radar plots show polyfunctionality of the (I) GFP-specific $CD4^+$ T cell response and the (J) GFP-specific $CD8^+$ T cell response in GFP-vaccinated mice at day 21. Geometric mean frequencies displayed. Mann-Whitney test with correction for multiple comparisons to compare the 15 polyfunctional populations between Ad5_GFP and Ad5/49K_GFP. $n = 5$ mice/group.

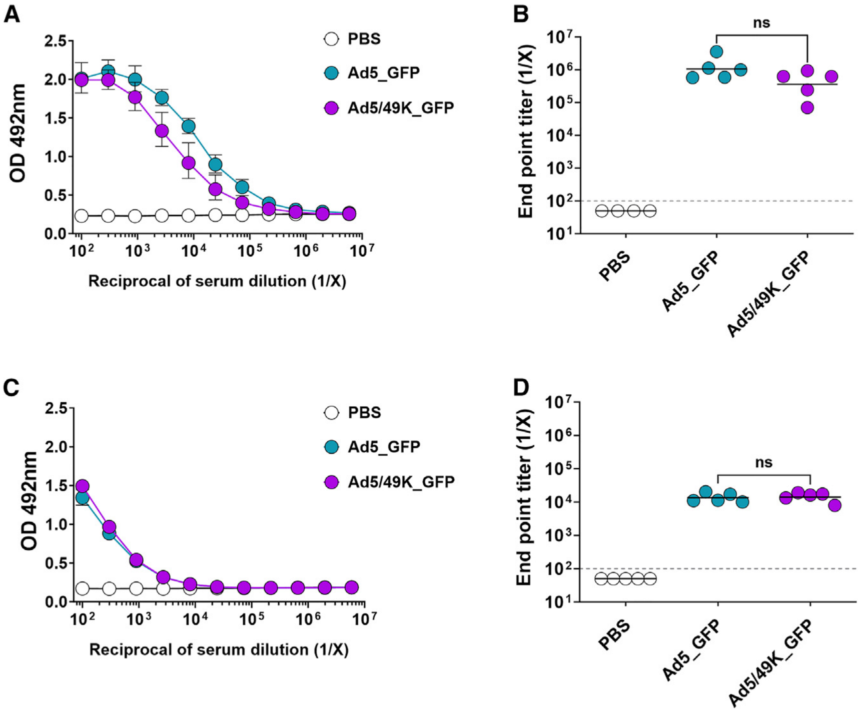

Figure 4. Ad5/49K vector induces comparable humoral immunogenicity to highly immunogenic Ad5 vector when administered as an i.m. vaccine to mice

ELISA measuring total serum IgG against recombinant protein or heat-inactivated Ad5 virus at day 21 following i.m. vaccination with 1 × 10^9 vp Ad5_GFP or 1 × 10^9 vp Ad5/49K_GFP. (A) Recombinant GFP ELISA using serial dilution of serum from 1:100 to 1:5,904,900. Geometric mean and geometric SD displayed. (B) Endpoint titer of anti-GFP serum IgG, as calculated using baseline optical density (OD) value of mean plus three standard deviations of naive mouse serum. (C) ELISA against Ad5 virus using serial dilution of serum from 1:100 to 1:5,904,900. Geometric mean and geometric SD displayed. (D) Endpoint titer of anti-Ad5 serum IgG, as calculated using baseline OD value of mean plus 3 SD of naive mouse serum. Dashed line represents starting dilution of 1:100. Lines represent geometric mean. Kruskal-Wallis test comparing Ad5_GFP to Ad5/49K_GFP only. ns, no significant difference. n = 4–5 mice/group.

antigen-specific CD8$^+$ T cells is key in the development of the latter. Ad5 and Ad5/49K vectors encoding full-length 5T4 were evaluated in a homologous prime-boost i.v. vaccination model in mice for induction and maintenance of 5T4-specific CD8$^+$ T cells. Ad5_5T4 and Ad5/49K_5T4 vaccine doses were administered at day 0 and day 28, alongside PBS-vaccinated control mice. Using sequential ICS analyses of 5T4 peptide-stimulated peripheral blood mononuclear cells (PBMCs), frequencies of 5T4-specific CD8$^+$ IFN-γ^+ cells were measured in the blood 7 days following priming vaccination with Ad5_5T4 and Ad5/49K_5T4 (Figure 5A), then at 1 week (Figure 5B) and 6 weeks (Figure 5C) post homologous boost vaccination. At each of these time points, Ad5/49K_5T4 induced elevated yet statistically non-significant frequencies of 5T4-specific CD8$^+$ T cells over Ad5_5T4 (geometric mean frequency of 5T4-specific CD8$^+$ IFN-γ^+ cells at day 7, 0.58% vs. 0.25%; at day 35, 0.21% vs. 0.15%; at day 70, 1.39% vs. 0.40% for Ad5/49K vs. Ad5). A time course summary of these responses revealed a significant increase in the adaptive response from day 35 to day 70 in the homologous Ad5/49K_5T4 group, suggesting 5T4-specific T cell responses were expanding in frequency over time post boost (Figure 5D). No such significant expansion was measured in the homologous Ad5_5T4 group.

At day 130, longevity of the 5T4-specific CD8$^+$ T cell response was evaluated using 5T4 peptide stimulation of splenocytes, followed by ICS. Importantly, the frequency of 5T4-specific CD8$^+$ IFN-γ^+ splenocytes following Ad5/49K_5T4 homologous vaccination was statistically higher than with homologous Ad5_5T4 vaccination (Figure 5E), indicating that Ad5/49K_5T4 vaccine was significantly more immunogenic than Ad5_5T4 and led to an increased accumulation of 5T4-specific T cells in peripheral tissues (geometric mean frequency of 5T4-specific CD8$^+$ IFN-γ^+ splenocytes at day 130, 0.39% vs. 0.10% for Ad5/49K vs. Ad5).

Ad5/49K vector exhibits enhanced transduction of primary human VSMCs

Enhanced *in vivo* immunogenicity is not a desirable characteristic for *in vivo* gene therapy applications of a vector; however, the field of *ex vivo* cell and gene therapy deploys vectors in the absence of major aspects of the immune system. One example of this is the potential gene therapy application to *ex vivo* saphenous vein during coronary artery bypass grafting. In terms of gene delivery, viral vectors with superior transduction activity of human VSMCs have potential for greater therapeutic efficacy in the coronary gene therapy setting, as well as dose sparing measures to minimize toxicity. The transduction profiles of Ad5_Luc and Ad5/49K_Luc were tested *in vitro* at a range of doses in VSMCs isolated from HSV. Ad5/49K vector exhibited statistically higher transduction efficiency of VSMCs than Ad5 vector when evaluated over a dose range of 1,000–10,000 vp/cell (Figure 6A, AUC statistical analyses in Figure S6A). The window of opportunity for *ex vivo* gene delivery during a coronary artery bypass graft procedure is approximately 30–60 min; therefore, a time course experiment was conducted to evaluate transduction efficiency when virus exposure time was limited to 10, 30, 60, or 180 min. Ad5/49K vector again exhibited statistically higher transduction of VSMCs compared with Ad5 across the four exposure times (Figure 6B, AUC statistical analyses in Figure S6B), with fold-change transduction of Ad5/49K over Ad5 measuring 210, 655, 230, and 533 at each respective time point (10, 30, 60, and 180 min). The 30 min vector exposure time represented

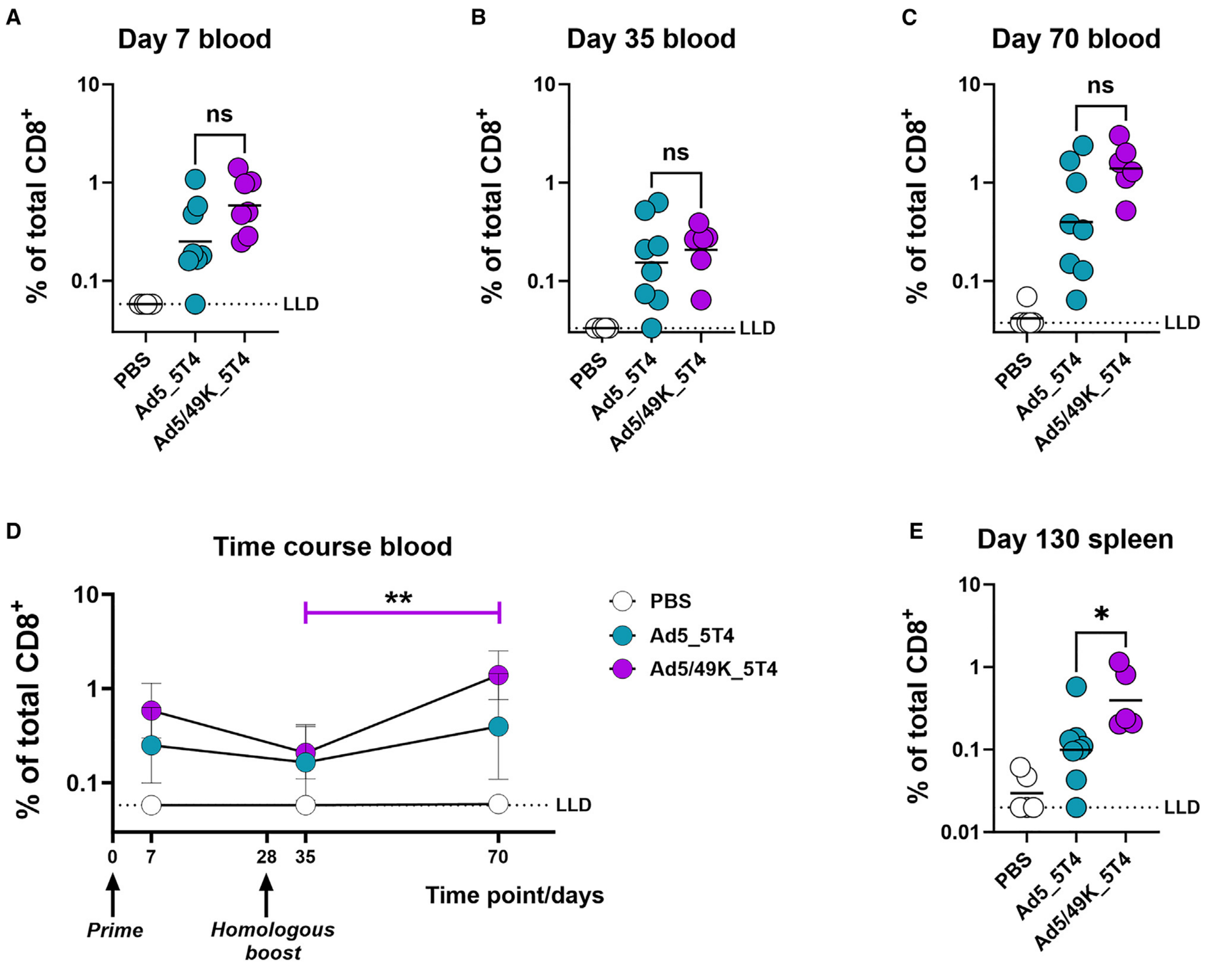

Figure 5. Ad5/49K vector induces enhanced CD8+ T cell immunogenicity compared with highly immunogenic Ad5 when administered as an i.v. cancer vaccine to mice

Flow cytometry with ICS on sequential PBMC samples collected following vaccination with PBS, or 5.41×10^{10} vp Ad5_5T4 or 5.41×10^{10} vp Ad5/49K_5T4 at day 0 and day 28. Frequencies of 5T4-specific $CD8^+IFN\gamma^+$ cells were measured in the blood following 5T4 peptide stimulation at (A) day 7, (B) day 35, and (C) day 70. Geometric mean displayed. Kruskal-Wallis test comparing Ad5_5T4 to Ad5/49K_5T4 only. ns, no significant difference. (D) Frequencies of 5T4-specific $CD8^+IFN\gamma^+$ PBMC summarized as a time course. Friedman paired test with Dunn's correction for multiple comparisons used between sequential mouse samples per Ad vaccine group. $**p < 0.01$. Geometric mean plus geometric SD displayed. (E) Splenocyte ICS measuring the frequency of 5T4-specific $CD8^+IFN\gamma^+$ cells following 5T4 peptide stimulation at day 130. Bar displayed at geometric mean. Kruskal-Wallis test comparing Ad5_5T4 to Ad5/49K_5T4 only. $*p < 0.05$. All responses represent the proportion of total parent $CD8^+$ T cells. LLD, lower limit of detection. n = 5–8 mice/group.

the greatest enhancement of Ad5/49K transduction over Ad5 vector, indicating the Ad5/49K vector would be more highly suited to the tight *ex vivo* gene therapy delivery window in the clinical setting between harvesting the HSV and coronary artery bypass grafting.

DISCUSSION

We report the activity of a chimeric adenoviral vector, Ad5/49K, for translational applications. The vector exhibits alterations to the Ad5 genome in a region of <600 base pairs of the fiber protein containing the primary receptor binding site. Although the receptor usage of Ad49 is unknown, its tropism is broad and includes cell lines devoid of all known Ad receptors, suggesting that a number of different cell entry mechanisms may be utilized by the virus.[34] Our data demonstrate Ad5/49K has improved ability to transduce VSMCs and DCs compared with Ad5 vector and enhanced immunogenicity as a vaccine vector, highlighting the potential downstream applications of this chimeric vector for both gene therapy and vaccine applications.

Using a model antigen (GFP) and a TAA (5T4), we demonstrate that Ad5 and Ad5/49K vectors induce strong T cell responses following

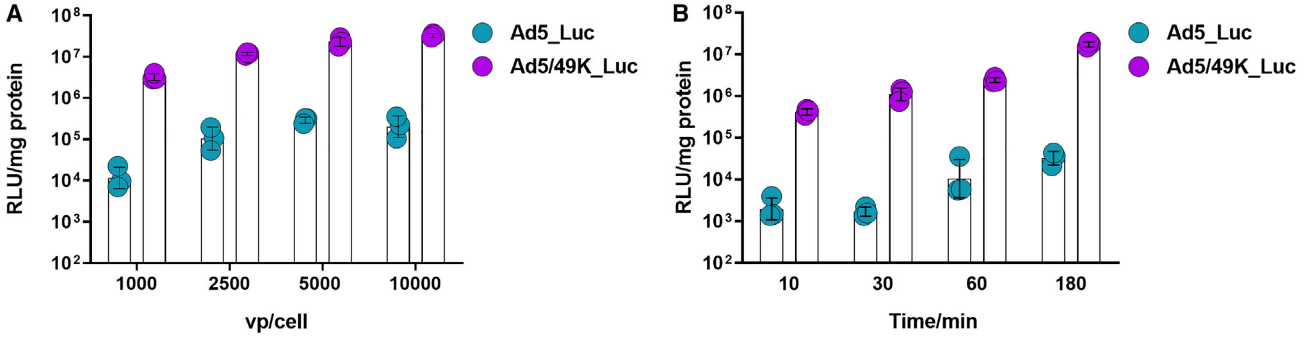

Figure 6. Ad5/49K vector exhibits increased transduction of primary human VSMCs

(A) Transduction of HSV smooth muscle cells with Ad5_Luc and Ad5/49K_Luc at 1,000, 2,500, 5,000, and 10,000 vp/cell. (B) Transduction of HSV smooth muscle cells with 2,500 vp/cell Ad5_Luc and Ad5/49K_Luc using virus exposure times of 10, 30, 60, and 180 min. Cell transduction measured by luciferase activity 48 h post infection, as determined by relative light units normalized to total cellular protein (RLU/mg). Geometric mean and geometric SD displayed. Statistical analyses using AUC outlined in Figure S6. *n* = 3/condition.

vaccination via the i.m. and i.v. routes. Following i.m. delivery, GFP-specific CD8$^+$ T cell responses were higher in frequency than GFP-specific CD4$^+$ T cells. This is in line with the literature, where i.m. Ad vectors consistently demonstrate dominance of the CD8$^+$ T cell response over the CD4$^+$ T cell response in mice.[50–52] The aim of the i.v. pre-clinical model was to evaluate the induction and maintenance of T cells in the context of a cytotoxic T cell cancer immunotherapy using a TAA. Robust 5T4-specific CD8$^+$ T cells were induced using the i.v. route of vaccine administration, with Ad5/49K_5T4 performing in a superior manner in terms of 5T4-specific CD8$^+$ T cell boosting and maintenance compared with Ad5_5T4. This suggests Ad5/49K is an improved vector candidate to take forward into an *in vivo* tumor challenge model. A previously published immunogenicity study of whole-serotype Ad49 vectored vaccine in mice reported lower CD8$^+$ T cell immunogenicity of this vaccine vector in comparison to Ad5 when administered via the i.m. route.[38] Pseudotyping of Ad49 fiber knob onto Ad5 vector gave comparable immunogenicity to Ad5 via the i.m. route yet superior immunogenicity over Ad5 via the i.v. route. Therefore, it is likely that a combination of the fiber knob pseudotyping *and* route of administration underpins the enhanced responses reported here; however, the difference in antigen used in our i.m. (GFP) and i.v. (5T4) vaccination experiments is a confounding variable.

Polyfunctionality of the antigen-specific T cell populations induced by i.m. Ad5_GFP and Ad5/49K_GFP vaccination is similar to that previously measured with Ad5.[51,53] These populations were also highly comparable between Ad5 and Ad5/49K, with the additional induction of a population of monofunctional CD4$^+$ IFN-γ^+ T cells following Ad5, but not Ad5/49K, vaccination. This population has previously been reported following pre-clinical Ad5 vaccination, with the population becoming more polyfunctional with addition of IL-2 and TNFα to the response following vaccination with non-Ad5-based vectors, such as Ad26.[53] This monofunctional CD4$^+$ T cell phenotype does not have a widespread association with protection in animal and human models of infectious disease or cancer, with such emphasis often placed on polyfunctional CD4$^+$ T cells[51,54–57] and CD8$^+$ T cells of both monofunctional[58] and polyfunctional phenotypes[59–62] during pre-clinical and clinical evaluation. Thus, the trend toward lower-frequency monofunctional CD4$^+$IFN-γ^+ cells following Ad5/49K vaccination compared to Ad5 does not represent a limitation of the chimeric vector, especially given the parity of the highly polyfunctional T cell phenotypes between the two vectors. Importantly, the most dominant antigen-specific CD4$^+$ and CD8$^+$ T cell populations induced by Ad5/49K were highly polyfunctional, lending its strong potential to such vaccine applications.

With high antibody titers induced by Ad5/49K against the encoded GFP transgene after a single i.m. vaccine dose, this vector has clear potential in infectious disease vaccines. Such robust and rapid induction of combined polyfunctional cellular immune responses and high-titer humoral immune responses after a single dose of vaccine are features deemed very desirable for such vaccines. Indeed, rapid induction of antigen-specific adaptive immune responses following a single shot of Ad vectored vaccine against infectious diseases in humans has previously been reported.[63–65] This is an especially attractive vaccine characteristic for rapid-response scenarios; for example, when the clinical application may require prompt ring vaccination to curtail an outbreak.[66] Single-shot vaccines are also highly suitable when follow-up for downstream dosing may be more challenging in resource-poor settings. Furthermore, mass vaccine-rollout campaigns also benefit from vaccine platforms that require only a single shot as part of their primary course, as seen with the U.K. Medicines and Healthcare products Regulatory Agency- (MHRA), European Medicines Agency- (EMA), and FDA-approved Ad26-based COVID-19 vaccine *Jcovden,* produced by Janssen. While direct comparisons are challenging to draw between our studies and those published in the literature due to differences in antigen and assay, the antibody titers induced by Ad5 and Ad5/49K in the data we report are of similar magnitude to responses reported in mice during the pre-clinical, single-shot evaluation of Jcovden.[67] The comparable Ad5 and Ad5/49K anti-GFP antibody data also suggest that the enhanced DC targeting

of Ad5/49K over Ad5 does not negatively impact the antigen-specific antibody response induced. Anti-vector antibody immunity generated toward Ad5 was also comparable between the two vectors, with responses against the hexon likely driving this response. This is consistent with the literature, where natural Ad infection induces antibodies against both the fiber and non-fiber capsid proteins, while antibodies directed toward non-fiber proteins, such as the penton base and hexon, tend to be more dominant following exposure to RD Ad5 vaccination.[68]

The importance of DCs in the priming of adaptive T cells has been extensively narrated in the context of both infectious diseases and in cancer immunotherapy.[69–72] *Ex vivo* manipulation of DCs can deploy proteins, peptides, or tumor lysates for DC loading[73–78] or involve the direct transfection of DCs with mRNA.[79–82] An *ex vivo* approach using mannose receptor targeting of antigen to APCs has already shown promise in patients with advanced epithelial malignancies.[83] However, such expensive and specialist *ex vivo* methodologies for cancer immunotherapy could be negated by direct *in vivo* delivery of the antigen using an Ad vector with improved DC transduction. We demonstrate enhanced transgene delivery in DCs with the Ad5/49K vector compared with Ad5. DCs do not generally express the primary Ad5 entry receptor,[42] CAR, so transduction is improved through the receptor usage of the pseudotyped Ad49 fiber knob. Comparatively, A549 cells with intermediate/high CAR expression exhibit more similar transduction by Ad5 and Ad5/49K vectors, suggesting a degree of specificity to the enhanced transduction measured in DCs.

Current Ad-based strategies that target DCs with pre-clinical success include targeting CD40 on DCs through Ad incorporation of a single domain antibody in the Ad fiber knob,[84] by expression of a large CD40L insert in the Ad fiber,[85] or through use of a bispecific adaptor molecule.[86] Our Ad5/49K approach does not require adenoviral expression of CD40L self-antigen and is unlikely to be hampered by Good Manufacturing Practice (GMP) production issues related to a mosaic viral capsid featuring such viral, bacteriophage, and human elements, thus highlighting a potential benefit of Ad5/49K vector over an existing DC targeting strategy.[86] Direct delivery of antigen to DC subsets in secondary lymphoid tissue has clear potential for improved antigen-specific immunogenicity. Indeed, such improved immunogenicity was measured in the direct comparison of Ad5 and Ad5/49K vaccination regimens in a mouse 5T4 immunogenicity model, where significantly higher frequencies of 5T4-specific $CD8^+$ T cells were measured after homologous i.v. Ad5/49K_5T4 vaccination, compared to homologous i.v. Ad5_5T4. Impressively, the chimeric vector performed well in terms of both T cell boosting and maintenance.

Finally, in the field of vascular gene therapy, delivery of therapeutic transgenes using viral (adenoviral, adeno-associated vectors, retroviral) and non-viral (plasmid and oligonucleotide delivery) approaches by subcutaneous, intra-arterial, i.m., and intramyocardial routes have shown limited success.[87] Despite many randomized controlled trials, none have proved beneficial for coronary artery disease, vein graft failure, or atherosclerosis, with the exception of lipid-lowering drugs.[88] For vein graft failure, the Project of *Ex-vivo* Vein graft Engineering via Transfection (PREVENT) III and PREVENT IV phase III randomized controlled trials delivered edifoligide, an oligonucleotide decoy targeting the E2F transcription factor to prevent progression of the cell cycle, using *ex vivo* pressure-mediated delivery to the vein graft. However, the results of both trials were negative.[89,90] We demonstrate the Ad5/49K vector transduces human VSMCs more efficiently than Ad5, and does so in the 30 min window of opportunity that is available during coronary artery bypass graft surgery, to deliver an *ex vivo* therapy to the vein graft. Consequently, Ad5/49K provides enhanced translational potential over pre-clinically evaluated Ad5 vector for the delivery of such genes to modify deleterious vascular remodeling.[91]

A clear dichotomy exists between the desire for immunogenic vectors for vaccine applications, where a strong immune response is desired against the vector's transient expression of the encoded antigen, in comparison to vectors for gene therapy applications, where low immunogenicity is conducive with efficient transgene delivery and minimal viral clearance. The latter is especially relevant in the setting of *in vivo* gene therapy, where an intact and present immune system plays a substantial role in the clearance of the virus. The Ad5/49K vector clearly exhibits increased transduction of a number of cell types, including DCs, which we hypothesize could underpin the increased immune responses measured against the transgene when deploying this vector in an *in vivo* vaccination setting. The application of the Ad5/49K vector to gene therapies that are delivered *ex vivo*, such as between vein harvesting and grafting in the coronary artery bypass graft procedure, are also highly suitable due to lack of immune cells in the vein graft during viral transduction. Subsequent grafting of transduced vein should not result in the downstream infection of immune cells (e.g., DCs), due to the non-replicating nature of the vector. Correspondingly, *in vivo* gene therapy applications may be less suited to the Ad5/49K vector, due to the enhanced DC transduction and immune stimulation demonstrated as part of the Ad5/49K vector's phenotype. This highlights the careful selection of Ad vectors for manipulation, evaluation, and downstream application.

In summary, we present the activity of a chimeric Ad vector based on Ad5 pseudotyped with the fiber knob of Ad49. We demonstrate the enhancement of this vector over Ad5 in multiple settings, highlighting the potential of Ad5/49K in vascular gene therapy, and in vaccine applications for both cancer and infectious diseases. Further research utilizing this platform should advance these pre-clinical studies and may yield other suitable downstream applications with unmet clinical needs.

MATERIALS AND METHODS

Generation of Ad5 and Ad5/49K viruses

Bacterial artificial chromosomes (BACs) encoding viral vectors, rendered replication deficient in the context of non-E1A complementing cells by deletion of the E1A and E3 regions, were generated using the recombineering method.[92] Transgenes (5T4, Luc, and GFP)

were codon optimised for human expression (Genewiz, Takeley, UK) and inserted under the control of the cytomegalovirus (CMV) promoter cassette using the *Escherichia coli* ribosomal protein S12 (rpsL) selection cassette (for Luc, GFP vectors) or the *Bacillus subtilis* levansucrase (SacB) selection cassette (for 5T4 vectors), previously described in the AdZ general recombineering protocol. Vector 1141 describes the CMV expression cassette (https://adz.cf.ac.uk/).[93–95] The Ad5/49K pseudotype was made using the same method. The fiber-knob pseudotype begins from the highly conserved T-L-W hinge motif in the fiber protein, which marks the transition from the fiber shaft to the fiber knob in human Ads. The nucleotide and amino acid sequences of the Ad49 fiber knob are described in GenBank: DQ393829.1 locus ABD52400.1 and were not altered or codon optimized.[96] The method of pseudotyping Ads post the T-L-W region and for this specific fiber knob have previously been outlined.[34,97–99]

Following generation of BACs encoding the desired modified Ad genome, BACs were transfected into T-REx-293 cells and monitored for the presence of cytopathic effect (CPE). Upon emergence of CPE, unpurified cellular material (~100 µL) was passaged into larger T-REx-293 cell cultures (5× T150 flasks) to expand the rescued virus. Following the presence of CPE throughout >70% of the flask, these cultures were harvested with all cells and media being centrifuged at 500 × *g* for 10 min, as described previously.[97] Supernatants were discarded and virus was purified from the cell pellet by the cesium chloride (CsCl) method.[100] This material was dialyzed into 10% glycerol, 10 mM Tris-HCl (pH7.8), 135 mM NaCl, and 1 mM $MgCl_2 \times 6H_2O$ buffer at 4°C overnight.

DNA was isolated from 200 µL of CsCl-purified Ad5_GFP and Ad5/49K_GFP virus using MinElute Virus amp spin kit (QIAgen, Hilde, Germany) as per the manufacturer's protocol, then DNA sequenced (Eurofin Genomics, Wolverhampton, UK). The sequences were processed by next-generation sequencing (NGS) analysis using Geneious software (Auckland, NZ) and assembled to a reference genome. DNA sequences were translated using SnapGene (San Diego, CA) and aligned using UniProt (Geneva, Switzerland).

MicroBCA virus titration

The Micro Bicinchoninic Acid Protein (BCA) Assay kit (Thermo Fisher Scientific, Waltham, MA) enabled total protein content of CsCl-purified virus preps to be calculated from a bovine serum albumin (BSA) standard curve. Viruses were measured in at least duplicate. The formula 1 µg of protein = 4×10^9 virus particles was used to convert to vp/mL titer.[47,48]

Nanoparticle size distribution

Purified viruses were diluted in PBS to obtain a concentration appropriate for nanoparticle analysis (1:500 Ad5_GFP and 1:100 Ad5/49K_GFP). Virus particles were acquired on a NanoSight NS300 instrument (Malvern Panalytical, Malvern, UK) using syringe pump speed of 100 (arbitrary units), and analyzed by Nanoparticle Tracking Analysis NTA 3.2 software (Malvern Panalytical, Malvern, UK).

Isolation of VSMCs from HSV

Saphenous vein segments surplus to surgical requirements were obtained from patients undergoing coronary artery bypass surgery (Research Ethics Committee number 14/EE/1097). HSV VSMCs were grown as described previously.[101] VSMCs were maintained in Dulbecco's Modified Eagle Medium (DMEM) growth medium (DMEM supplemented with 100 µg/mL of penicillin, 100 IU/mL streptomycin, 2 mM L-glutamine, and 10% [v/v] fetal calf serum [FCS]).

VSMC *in vitro* transduction assay

Cells were seeded at a density of 2×10^4 cells per well in a 96-well plate. After 24 h, cells were infected with virus at doses of 1,000, 5,000, and 10,000 vp per cell in a total volume of 100 µL of serum-free medium and incubated for 3 h. The medium was removed and replaced with 200 µL of complete medium (Roswell Park Memorial Institute [RPMI] 1640 medium supplemented with 200 µM Glutamax, 10% [v/v] FCS, 100 U/mL penicillin, 100 µg/mL streptomycin and 10% [v/v] autologous supernatant) and cultured for an additional 45 h. For luciferase assays, cells treated with Ad5 and Ad5/49K (encoding luciferase) were lysed in 1× Cell Culture Lysis Buffer (Promega, Southampton, UK) and frozen at −70°C. The cells were thawed and 20 µL of cells was mixed with 100 µL of luciferase assay reagent in a white 96-well plate. Luc activity in relative light units (RLU) was measured immediately using a multimode plate reader (FLUOstar Omega, BMG Labtech, Aylesbury, UK). Samples were normalized for total protein content as measured by bicinchoninic acid assay to give RLU per milligram of protein, using the plate reader as described above. Experiments were performed in triplicate.

Derivation of human DCs

An apheresis cone supplied by the Welsh Blood Service was washed with PBS plus heparin sodium (1,000 U/mL, Wockhardt, Mumbai, India) and the blood layered over Histopaque (Sigma 10771), then centrifuged at 2,000 rpm for 20 min with no brake to obtain the PBMCs. The PBMC layer was removed and washed in PBS, and $CD14^+$ monocytes isolated by magnetic activated cell sorting (MACS) using $CD14^+$ microbeads and LS columns. The purity of the $CD14^+$ sample was assessed by flow cytometry with a CD14 antibody. Then, purified $CD14^+$ monocytes were seeded into un-treated cell culture plates, RPMI was supplemented with 10% FCS, 2 mM L-glutamine, IL-4 (100 ng/mL, Peprotech, Altrincham, UK), granulocyte-macrophage colony-stimulating factor (GM-CSF) (100 ng/mL, Peprotech, Altrincham, UK), and β-mercaptoethanol (50 nM, Gibco, Waltham, MA). Media and supplement changes were carried out every 3 days, excluding β-mercaptoethanol, which was added on the day of isolation only. DCs were phenotyped 6 days following purification by staining with anti-CD14 PECy7 (1:100, eBioscience), anti-CD1a FITC (1:100, BD Pharmingen, San Diego, CA), and anti-DC-SIGN PE (1:100, BD Biosciences, Franklin Lakes, NJ) in PBS for 15 min at 4°C, washing with PBS, fixation in 4% paraformaldehyde, followed by acquisition on a C6 Accuri flow cytometer (Beckton Dickinson, Franklin Lakes, NJ). Cells that were CD14-low, DC-SIGN-high, and CD1a-high were considered successful human

monocyte-derived DC (hMo-DC) cultures. Validation of hMo-DCs using surface marker expression is outlined in Figure S1.

Human DC transduction assay

hMo-DC cultures were used on the day of culture harvest (day 6). Cells were seeded at 25,000/well in V-bottom plates in serum-free RPMI medium supplemented with 100 μg/mL penicillin-streptomycin and 2 mM L-glutamine (denoted R0). Virus was serially diluted in R0 and added to cells in increasing virus particle:cell ratios from 100 vp/cell to 5,000 vp/cell and then incubated at 37°C with 5% CO_2 for 2 h. Cells were washed with PBS (1,600 rpm, 4 min) before resuspending in 150 μL of RPMI medium supplemented with 10% FBS, 100 μg/mL penicillin-streptomycin, and 2 mM L-glutamine (denoted R10). Cells were incubated at 37°C with 5% CO2 for a further 22 h. Cells were then washed with PBS (1,600 rpm, 4 min) and stained with Far Red LIVE/DEAD cell stain (Life Technologies, Waltham, MA) at 1:1,250 diluted in fluorescence-activated cell sorting (FACS) buffer (PBS + 5% FBS) for 30 min at 4°C. Cells were then washed twice with FACS buffer, fixed in 3.7% formaldehyde for 20 min at 4°C, washed twice with FACS buffer, then resuspended in FACS buffer for acquisition on a C6 Accuri flow cytometer (Beckton Dickinson, Franklin Lakes, NJ) via FL-1 for GFP and FL-4 for Far Red. Minimum of 3,000 live DCs acquired per sample. Representative gating strategy for analysis is outlined in Figures S3A and S3B. Single-color compensation controls were used to calculate compensation before analysis in FlowJo v10.8.1 (BD Life Sciences, Franklin Lakes, NJ). Percentage GFP transduction of live cells was reported, with background fluorescence subtracted using uninfected cell controls. Geometric mean fluorescence intensity of GFP^+ population was also reported.

Mouse DC isolation

Mice were euthanized with CO_2 and femurs and tibiae dissected from hind limbs. Bones were sterilized for 1 min with 70% ethanol and then cut at both ends with scissors. Bone marrow was flushed into a 50 mL conical tube, with a 25-gauge needle and a 5 mL syringe, using 10 mL of RPMI medium. The suspension was vortexed gently and pipetted up and down to homogenize, then centrifuged for 5 min at 350 × *g*. The pellet was resuspended in 5 mL of RPMI. The suspension was passed through a 40 μm cell strainer and centrifuged again, then the pellet was resuspended in 1 mL of in-house red blood cell (RBC) lysis buffer and incubated for 3 min. The lysis was stopped by adding 10 mL of PBS, the solution was transferred to a tube containing 1 mL of FCS, and it was centrifuged at 1,500 rpm for 5 min. The pellet was washed twice with RPMI buffer and finally resuspended in 1 mL of RPMI medium supplemented with 10% heat-inactivated FCS, 100 μg/mL penicillin-streptomycin, 2 mM L-glutamine, 5 mL of Minimum Essential Medium (MEM), 5 mL of HEPES, 50 μM β-mercaptoethanol, and 20 ng/mL of GM-CSF (Peprotech, denoted cDC media). Cells were plated at a density of 4×10^6/mL in sterile petri dishes, in 10 mL of cDC medium. Cells were incubated at 37°C with 5% CO_2. On day 2, 10 mL of fresh cDC medium was added. On day 4, 10 mL was taken from each plate, centrifuged at 1,500 rpm for 5 min, and resuspended in 10 mL of fresh cDC medium.

Mouse DC transduction assay

Bone marrow-derived DCs were used on day 7. Cells were seeded at 2×10^5 cells per well in flat-bottom 96-well plates in cDC medium and incubated overnight. Next day, cells were washed by centrifugation with PBS (1,500 rpm, 5 min) and 100 μL of virus, which was serially diluted in serum-free RPMI, was added in increasing virus particle:cell ratio, at 100, 500, 1,000, 2,500, and 5,000 vp/cell. Cells were incubated at 37°C with 5% CO_2 for 3 h, then centrifuged (1,500 rpm, 5 min) and resuspended in complete growth medium R10. Cells were incubated at 37°C with 5% CO_2 for a further 24 h. Cells were transferred to V-bottom plates, washed with PBS, and stained with LIVE/DEAD Aqua Zombie cell stain (BioLegend, San Diego, CA) diluted at 1:500 in PBS for 10 min at room temperature (RT). Cells were then washed with PBS and FACS buffer, fixed in 3.7% formaldehyde for 30 min at RT, washed twice with FACS buffer, and resuspended in 200 μL of FACS buffer for acquisition on an Attune NxT Flow Cytometer (Thermo Fisher Scientific, Waltham, MA). Single-color compensation controls were used to calculate compensation before analysis in FlowJo 10.9.0 (BD Life Sciences, Franklin Lakes, NJ). A representative gating strategy for analysis is outlined in Figures S3C and S3D. Percentage GFP transduction of live cells was reported, with background fluorescence subtracted using uninfected cell controls. Geometric mean fluorescence intensity of the GFP^+ population was also reported.

A549 cell transduction assay

A549 cells were seeded in 96-well plates at a density of 15,000 cells/well in complete medium consisting of RPMI containing 10% FBS, 100 μg/mL penicillin-streptomycin, and 2 mM L-glutamine (denoted R10). After 24 h incubation at 37°C with 5% CO_2, cells were washed with PBS, then infected with viruses at doses of 1,000, 5,000, and 10,000 vp per cell in a total volume of 50 μL of serum-free RPMI medium supplemented with 100 μg/mL penicillin-streptomycin and 2 mM L-glutamine (denoted R0), and incubated for 3 h at 37°C with 5% CO_2. The virus was removed and replaced with 200 μL of complete R10 medium for a further 45 h incubation as above. After the total 48 h incubation, medium was removed and cells washed with 200 μL of PBS and treated with 50 μL of trypsin/EDTA at 37°C for 10 min before recovery in 100 μL of FACS buffer (PBS + 5% FBS) and transfer to a V-bottom plate. Cells were washed in 200 μL of PBS (1,600 rpm, 5 min), then stained with 50 μL of 1:2,000 Far Red Amine Reactive LIVE/DEAD Fixable Dye (Life Technologies, Waltham, MA) for 30 min at 4°C. Cells were washed twice in FACS buffer (1,600 rpm, 5 min), fixed in 2% formaldehyde for 20 min at 4°C, then washed twice more in FACS buffer before resuspending in FACS buffer for acquisition on a C6 Accuri flow cytometer (Beckton Dickinson, Franklin Lakes, NJ) via FL-1 for GFP and FL-4 for Far Red. A minimum of 3,000 live A549 cells were acquired per sample. Single-color compensation controls were used to calculate compensation before analysis in FlowJo v10.4.2 (BD Life Sciences, Franklin Lakes, NJ). Samples were analyzed using a gating strategy similar to that used for human DCs. Percentage GFP transduction of live cells was reported, with background subtraction of autofluorescence from

uninfected cell controls. Geometric mean fluorescence intensity of GFP^+ population was also reported.

Mouse vaccination studies

The i.m. mouse vaccination studies were conducted at the Icahn School of Medicine at Mount Sinai (ISMMS) Hospital and were approved by the Icahn School of Medicine at Mount Sinai Institutional Animal Care and Use Committee (IACUC-2017-0170). Animal studies adhered to the Animal Research: Reporting of *In Vivo* Experiments (ARRIVE) guidelines.[102] Female BALB/cJ mice (Jackson Laboratory) aged 7 weeks received 1×10^9 vp of either Ad5_GFP or Ad5/49K_GFP vaccine diluted in sterile PBS in a total volume of 50 μL. Control animals received 50 μL of PBS (Life Technologies, Waltham, MA). $n = 5$ mice per group. A pre-vaccination blood sample was obtained from each animal by submandibular bleeding into Microvette CB300 capillary blood collection tubes with clot activator (Sarstedt, Nümbrecht, Germany) 4 days prior to vaccination and used as pooled naive serum for ELISA assays. Maximal blood sampling throughout the experiment did not exceed recommended guidelines per total blood volume (TBV), as established by the National Centre for the Replacement, Refinement and Reduction of Animals in Research (NC3Rs): a maximum of <10% on any single occasion and <15% TBV within 28 days. At day 21 following vaccination, mice were euthanized by exsanguination via cardiac puncture under terminal anesthesia of intraperitoneal xylazine and ketamine, with blood collected into Microtainer tubes with clot activator (BD Biosciences, Franklin Lakes, NJ) for serum separation. Death was confirmed by cervical dislocation, and spleens dissected into 1 mL of cold PBS. For serum separation, Microtainer tubes with clot-activator tubes were then centrifuged at 15,000 × *g* for 10 min, serum aliquoted, and stored at −20°C. Spleens were mechanically disrupted through a 40 μm cell strainer into PBS and then pelleted by centrifugation at 450 × *g* for 5 min. Supernatants were discarded and splenocytes resuspended in ammonium-chloride-potassium (ACK; 0.15 M NH_4Cl, 10 mM $KHCO_3$, and 100 mM EDTA-Na_2 in water) lysing buffer and incubated for 5 min at RT, vortexing at the start and end of incubation. PBS was added per sample to stop the lysis, samples were pelleted by centrifugation at 500 × *g* for 5 min, and the supernatant was discarded. Samples were resuspended in R10, and cell debris clump was removed and then re-pelleted by centrifugation at 500 × *g* for 5 min. Splenocytes were resuspended in R10 for subsequent manual counting in trypan blue and then resuspended to 30×10^6 live splenocytes/mL, ready for splenocyte evaluation using flow cytometry with ICS.

The i.v. mouse studies were conducted at Cardiff University, under the UK Home Office License PPL 30/3428. Mice were vaccinated i.v. at day 0 and day 35 with 5.41×10^{10} vp of Ad5_5T4 or Ad5/49K_5T4, or equivalent 50 μL volume of PBS diluent. Flow cytometry with ICS was performed on PBMCs at day 7, day 35, and day 70 following tail vein bleeds. T cell responses in the spleens were assessed 130 days post vaccination following schedule 1 euthanasia of mice. For tail vein blood collection, mice were placed in a heating chamber at 35°C for 20 min. Then, animals were placed in a restraining tube and local anesthetic (ethyl chloride, Vidant Pharma, Cambridge, UK) was sprayed on the tail before collecting blood from the lateral tail vein. Blood was collected into lithium-heparin-coated tubes for capillary blood collection (Microvette CB300 LH, Sarstedt, Nümbrecht, Germany). For splenocyte analysis, spleens were dissected from euthanized mice and mashed twice through a 70 μm nylon cell strainer (Thermo Fisher Scientific, Waltham, MA) using a plunger top of a 2 mL syringe to obtain a single-cell suspension, with PBS washes in between. The suspension was centrifuged for 5 min at 1,500 rpm and the pellet was resuspended in 3 mL of RBC lysis buffer and incubated for 5 min. The lysis was stopped with 3 mL of PBS, the suspension centrifuged for (8,000 rpm, 5 min), and the pellet resuspended in R10 buffer. Splenocytes were quantified using a LUNA cell counter (Logos Biosystems, Lille, France) and resuspended at 1×10^7 splenocytes/mL for stimulation and flow cytometry with ICS.

Flow cytometry with ICS

For detection of GFP-specific T cells, splenocytes from GFP-vaccinated mice were stimulated in R10 medium containing anti-mouse CD28 (1:1,000, BD Biosciences, Franklin Lakes, NJ), brefeldin A (1:1,000, BD Biosciences), Monensin (1:1,000, BD Biosciences, Franklin Lakes, NJ), and anti-mouse CD107a-PE (1:200, BioLegend, San Diego, CA) for 6 h at 37°C in 5% CO_2. Stimulations consisted of either 1 μg/mL of pooled GFP peptides (JPT Peptides, Berlin, Germany) in R10 medium or an equivalent volume of R10 medium with DMSO as a negative control. Separate splenocytes underwent stimulation with a combination of 0.5 μg/mL phorbol 12-myristate 13-acetate (PMA; Sigma-Aldrich, St. Louis, MO) and 1 μg/mL ionomycin (Sigma-Aldrich, St. Louis, MO) as a positive control. After stimulation, plates were stored at 4°C overnight protected from light. Cells were then pelleted by centrifugation at 500 × *g* for 5 min in an Eppendorf 5810R centrifuge and then incubated with Fc block (1:100 BD Biosciences, Franklin Lakes, NJ) for 10 min at 4°C. Cells were washed in FACS buffer and incubated with surface-staining cocktail for 30 min at 4°C protected from light (Table S1). After incubation, cells were washed in FACS buffer and then incubated in fixation/permeabilization buffer (BD Biosciences) for 10 min at 4°C. Cells were washed in 1× permeabilization buffer (BD Biosciences, Franklin Lakes, NJ) then incubated with the intracellular staining cocktail for 30 min at 4°C protected from light (see Table S1). Samples were washed twice in 1× permeabilization buffer and once in FACS buffer, then resuspended in FACS buffer. Samples were acquired on an LSRII flow cytometer (BD Biosciences, Franklin Lakes, NJ) using FACSDiva v7.03 (BD Biosciences, Franklin Lakes, NJ) with the relevant single-fluorochrome compensation controls (UltraComp eBeads and ArC Amine Reactive Beads, Thermo Fisher Scientific, Waltham, MA), and photon multiplier tube voltages were set by daily acquisition of Cytometer Setup and Tracking beads (BD Biosciences, Franklin Lakes, NJ).

For detection of 5T4-specific $CD8^+$ T cells, PBMCs and splenocytes from 5T4-vaccinated mice were stimulated with peptides spanning mapped immunodominant 5T4 epitopes at a final concentration of 3 μg/mL for 6 h at 37°C in 5% CO_2 in the presence of brefeldin A

(2 μg/mL, BD Biosciences, Franklin Lakes, NJ). Cells were then stained with LIVE/DEAD Zombie Aqua dye (BioLegend, San Diego, CA) and incubated with Fc block. Cells were subsequently stained with surface-staining cocktail for 15 min at RT protected from light (see Table S2) and fixed in 4% paraformaldehyde. Next day, cells were permeabilized with saponin buffer (PBS, 2% FCS, 0.05% sodium azide, and 0.5% saponin) for 10 min at RT in order to perform ICS for 10 min at RT (see Table S2). Samples were washed in saponin buffer, then washed twice in FACS buffer, and finally resuspended in 200 μL of FACS buffer for acquisition on an Attune NxT Flow Cytometer (Thermo Fisher Scientific, Waltham, MA). Compensation was performed using anti-rat/hamster antibody-capture beads (BD Comp Beads, BD Biosciences, Franklin Lakes, NJ) with relevant fluorochromes. Attune Performance Tracking Beads (Thermo Fisher Scientific, Waltham, MA) were used at each time point to ensure the optimal instrument performance.

Flow cytometry with ICS data analysis

For ICS of i.m. GFP-vaccinated mice, >54,588 $CD4^+$ and >22,759 $CD8^+$ T cells were acquired per splenocyte test sample. Data were analyzed using FlowJo v10.4.2 (BD Life Sciences, Franklin Lakes, NJ) gating on lymphocytes, singlets, live cells, $CD3^+$, $CD4^+$, or $CD8^+$ and then assessed for IFN-γ, IL-2, TNFα, and CD40L responses and for CD107a expression. Responses in autologous DMSO negative controls were subtracted from GFP-stimulated and PMA/ionomycin-stimulated responses, with GFP-specific responses reported as frequency of positive cells per parent $CD4^+$ or $CD8^+$ T cell population. All PMA/ionomycin-stimulated splenocytes passed a >1% cytokine positive-response threshold, and all DMSO samples passed a <0.2% cytokine negative-response threshold. Boolean gating permitted export of polyfunctional $CD4^+$ or $CD8^+$ T cell responses, which were subject to the equivalent subtraction of autologous DMSO responses. Compensation was calculated in FlowJo based on single-fluorochrome compensation bead controls.

For ICS of i.v. 5T4-vaccinated mice, >1,730 $CD8^+$ T cells from blood and >5,000 $CD8^+$ splenocytes were acquired per test sample. Data were analyzed using FlowJo v10.8.1 (BD Life Sciences, Franklin Lakes, NJ) gating on lymphocytes, singlets, live cells, and $CD8^+$ and then assessed for cytokine production. Responses in autologous (media) negative controls were subtracted from 5T4-stimulated responses, with 5T4-specific responses reported as frequency of positive cells per parent $CD8^+$ T cell population. All negative control samples passed a <0.2% cytokine negative-response threshold or were excluded from the analyses. Compensation was calculated in FlowJo based on single-fluorochrome compensation bead controls.

A sample gating strategy is provided (Figures S4A–S4C, S5A–S5B) for flow cytometry with ICS of i.m. and i.v. vaccination studies, respectively.

Total IgG ELISA

For anti-GFP ELISA, Immulon 4HBX flat bottom 96-well plates (Thermo Fisher Scientific, Waltham, MA) were coated overnight at 4°C with recombinant EGFP protein (Abcam, Cambridge, UK) at 1 μg/mL diluted in 50 μL of 50 mM carbonate Na_2CO_3 buffer (Sigma-Aldrich, St. Louis, MO). The following day, plates were washed using 1× PBS (Life Technologies, Waltham, MA) containing 0.1% Tween 20 (Millipore, Burlington, MA, denoted TPBS) and blocked with 200 μL of blocking buffer: PBS containing 1% (w/v) BSA (Sigma-Aldrich, St. Louis, MO) for at least 1 h at RT. After washing with TPBS, a 1:100 dilution of mouse sera was added to the plate in duplicate and a 3-fold serial dilution in blocking buffer performed, resulting in a final volume of 100 μL/well. Plates were incubated for 2 h at RT on an orbital shaker. A mouse monoclonal antibody (mAb) to EGFP (Abcam, Cambridge, UK) was used as a positive control, and an isotype control (mouse monoclonal IgG_1; Abcam) was used as a negative control on every plate. Additional controls on each plate included naive, unvaccinated mouse sera and secondary antibody-only controls. After washing with TPBS, 100 μL of goat anti-mouse IgG horseradish peroxidase (HRP)-conjugated secondary Ab (1:5,000, Millipore, Burlington, MA) added to the plate. After a 1 h incubation at 37°C, the plate was washed and developed using 100 μL of SigmaFast *o*-phenylenediamine dihydrochloride (OPD; Sigma-Aldrich, St. Louis, MO) tablets diluted in water. Development was stopped with 50 μL of 3 M HCl after 6 min. Plates were read for optical density (OD) at 492 nm.

For anti-Ad5 ELISA, 96-well Immulon 4 HBX 330 μL flat bottom plates (Thermo Fisher Scientific, Waltham, MA) were coated with 200 ng/mL heat-inactivated (56°C for 30 min) empty Ad type 5 diluted in 100 μL of 50 mM carbonate Na_2CO_3 buffer (Sigma-Aldrich, St. Louis, MO) overnight at 4°C. The next day, the plates were washed using a 1× PBS/0.1% Tween 20 (TPBS) solution (PBS, Corning, Corning, NY; and Tween 20, Sigma-Aldrich, St. Louis, MO) and blocked with 200 μL of 1× PBS/1% BSA (PBS, Thermo Fisher Scientific, Waltham, MA; and BSA, MP Biomedicals, Irvine, CA) blocking buffer for 1–1.5 h on a rocking shaker at RT. After washing with TPBS, a 1:100 dilution of mouse sera was added to the plate (in duplicate) and a 3-fold serial dilution in blocking buffer was performed, resulting in final volume of 100 μL/well. A positive-control mAb, anti-Ad (B025/AD51) (Abcam, Cambridge, UK), which binds to the hexon polypeptide across all Ad serotypes, was used on all plates at a final concentration of 10 μg/mL. A matched isotype control, clone no. MOPC-173 mouse IgG2a (Abcam, Cambridge, UK), was used as the negative control on each plate. Unvaccinated mouse sera and secondary antibody only served as additional controls on every plate. Plates were incubated for 2 h at RT on a rocking shaker. After washing, 100 μL of 1:5,000 dilution of goat anti-mouse IgG HRP-conjugated secondary antibody (Millipore, Burlington, MA) was added to the plate then incubated at 37°C for 1 h. After the incubation, the plate was washed and developed using 100 μL of SigmaFast OPD (Sigma-Aldrich, St. Louis, MO) tablets diluted in water. Development was stopped after 8 min with 50 μL of 3M HCl. Plates were read for OD at 492 nm.

The ELISA baseline was defined as the mean plus 3SD of naive sera OD values across all plates, which were evaluated in a single run.

The mean of test sera duplicate values was calculated for each dilution. Endpoint titers were calculated using GraphPad Prism v9.5.1 (Dotmatics, Boston, MA) and represent the X intercept with the defined baseline (naive mean plus 3SD). Values below the limit of detection were estimated to be at half the highest input dilution (i.e. 1:50).

Statistical analyses

All statistical analyses were performed using GraphPad Prism v9.5.1 (Dotmatics, Boston, MA). Data were tested for a normal distribution using D'Agostino and Pearson test and were not deemed normally distributed, so non-parametric tests were deployed: Mann-Whitney test for two vaccine groups, with Holm-Šídák correction for multiple comparisons applied where multiple polyfunctional T cell groups were compared; Kruskal-Wallis test for three vaccine group analyses, with comparison between Ad5 and Ad5/49K defined; Friedman paired test for sequential (paired) mouse samples per vaccine group across three time points, with Dunn's correction for multiple comparisons. Friedman paired analysis excludes one mouse with missing time-point data. All data points are graphed. $*p < 0.05$, $**p < 0.01$, $***p < 0.001$, $****p < 0.0001$. AUC analyses were performed to determine area under dose-response data (not curve fitted). Unpaired t tests were used to compare AUCs (mean, SEM) between the two vaccine conditions. Degrees of freedom (df) were calculated as {df = # data points − # groups}. Sample size (n) was determined as: {n = df + 1}.

DATA AND CODE AVAILABILITY

Raw data and analyses that support the findings of this study can be made available by the corresponding author upon reasonable request and under a data transfer agreement.

SUPPLEMENTAL INFORMATION

Supplemental information can be found online at https://doi.org/10.1016/j.omtm.2024.101308.

ACKNOWLEDGMENTS

Schematic figures created with BioRender.com. Mouse immunogenicity experiments were performed at Cardiff University and at ISMMS Hospital. We therefore thank the Joint Biological Services Unit at Cardiff University and the Center for Comparative Medicine and Surgery at the ISMMS for their assistance. We additionally acknowledge laboratory support staff across the institutions, and we specifically thank the members of the Flow Cytometry Core at ISMMS.

C.M.B. was funded by Life Sciences Hub Wales Accelerate grant (reference PR-0308), Wellcome Trust Institutional Strategic Support Fund Fellowship (reference AC1170IF04), Wales Cancer Research Centre CReSt Catalytic funding and Higher Education Funding Council for Wales (HEFCW). S.L.H.-C. was funded by a Cancer Research UK ECMC center award (C7838/A25173). M.W. was supported in part by grant MR/N0137941/1 for the GW4 BIOMED MRC DTP, awarded to the Universities of Bath, Bristol, Cardiff, and Exeter from the Medical Research Council (MRC)/UKRI. M.M. was funded by the Wales Cancer Research Centre and the Experimental Cancer Research Centre (C7838/A25173). J.A.D. was funded by a Cancer Research UK Biotherapeutic Programme grant to A.L.P. (reference C52915/A29104). E.S. was supported by funding from the MRC (MR/S00971X/1). A.T.B. was funded by a Tenovus Cancer Care PhD studentship to A.L.P (reference PhD2015/L13). L.S. was funded by a Harry Smith Vacation Studentship from the Microbiology Society (reference GA002539) awarded to C.M.B. L.K. was funded by institutional seed funding (University of Maryland, Baltimore) awarded to L.C. L.E.K.-J. was funded by a Kidney Research UK Project Grant (RP_008_20210729) and Wellcome Trust Discovery Award (204870/Z/16/Z). K.M. was funded by Wales Cancer Research Centre CReSt Catalytic funding. G.R. was funded by institutional seed funding (University of Maryland, Baltimore) awarded to L.C. S.J.G. was funded by Higher Education Funding Council for England (HEFCE). L.M.B. was funded by a Cancer Research UK Biotherapeutic Programme grant to A.L.P. (reference C52915/A29104). R.J.S was funded by Wellcome Trust (226615/Z/22/Z) and Higher Education Funding Council for Wales (HEFCW). L.C. was funded by NIAID (reference R21AI157606). I.R.H. was funded by a Wellcome Trust Senior Research Fellowship (207503/Z/17/Z) and MRC Project grant (MR/X00922X/1). A.L.P. was funded by the HEFCW.

AUTHOR CONTRIBUTIONS

Conceptualization, C.M.B. and A.L.P.; data curation, C.M.B., S.L.H.-C., and M.M.; formal analysis, C.M.B., S.L.H.-C., K.M., and A.L.P.; funding acquisition, C.M.B., R.J.S., I.R.H., and A.L.P.; investigation, C.M.B., S.L.H.-C., M.W., M.M., L.S., L.K., G.R., L.M.B., and L.C.; methodology, C.M.B., S.L.H.-C., M.W., J.A.D., E.S., A.T.B., L.S., L.K., L.E.K.-J., G.R., S.J.G., L.M.B., R.J.S., L.C., I.R.H., and A.L.P.; project administration, J.A.D. and A.L.P.; resources, C.M.B., J.A.D., A.T.B., S.J.G., R.J.S., L.C., I.R.H., and A.L.P.; supervision, C.M.B., J.A.D., L.M.B., R.J.S., I.R.H., and A.L.P.; validation, C.M.B., M.M., E.S., A.T.B., and L.E.K.-J.; visualization, C.M.B., M.M., L.E.K.-J., and A.L.P.; writing – original draft, C.M.B., S.L.H.-C., and A.L.P.; writing – review & editing, C.M.B., S.L.H.-C., M.W., M.M., J.A.D., E.S., A.T.B., L.S., L.E.K.-J., K.M., G.R., S.J.G., L.M.B., R.J.S., L.C., I.R.H., and A.L.P.

DECLARATION OF INTERESTS

A.L.P. is founder and CSO of Trocept Therapeutics (part of Accession Therapeutics Ltd.), who are developing unrelated viral platforms for oncolytic virotherapy applications.

REFERENCES

1. HAdV Working Group (accessed on 4 January 2024). http://hadvwg.gmu.edu/.
2. Lynch, J.P., and Kajon, A.E. (2016). Adenovirus: Epidemiology, Global Spread of Novel Serotypes, and Advances in Treatment and Prevention. Semin. Respir. Crit. Care Med. *37*, 586–602. https://doi.org/10.1055/s-0036-1584923.
3. Ricobaraza, A., Gonzalez-Aparicio, M., Mora-Jimenez, L., Lumbreras, S., and Hernandez-Alcoceba, R. (2020). High-capacity adenoviral vectors: expanding the scope of gene therapy. Int. J. Mol. Sci. *21*, 3643.
4. O'Hara, G.A., Duncan, C.J.A., Ewer, K.J., Collins, K.A., Elias, S.C., Halstead, F.D., Goodman, A.L., Edwards, N.J., Reyes-Sandoval, A., Bird, P., et al. (2012). Clinical

assessment of a recombinant simian adenovirus ChAd63: a potent new vaccine vector. J. Infect. Dis. *205*, 772–781. https://doi.org/10.1093/infdis/jir850.

5. Shiver, J.W., Fu, T.-M., Chen, L., Casimiro, D.R., Davies, M.-E., Evans, R.K., Zhang, Z.-Q., Simon, A.J., Trigona, W.L., Dubey, S.A., et al. (2002). Replication-incompetent adenoviral vaccine vector elicits effective anti-immunodeficiency-virus immunity. Nature *415*, 331–335. https://doi.org/10.1038/415331a.

6. Zahn, R., Gillisen, G., Roos, A., Koning, M., van der Helm, E., Spek, D., Weijtens, M., Grazia Pau, M., Radošević, K., Weverling, G.J., et al. (2012). Ad35 and ad26 vaccine vectors induce potent and cross-reactive antibody and T-cell responses to multiple filovirus species. PLoS One *7*, e44115. https://doi.org/10.1371/journal.pone.0044115.

7. Barnes, E., Folgori, A., Capone, S., Swadling, L., Aston, S., Kurioka, A., Meyer, J., Huddart, R., Smith, K., Townsend, R., et al. (2012). Novel adenovirus-based vaccines induce broad and sustained T cell responses to HCV in man. Sci. Transl. Med. *4*, 115ra1.

8. O'Hara, G.A., Duncan, C.J.A., Ewer, K.J., Collins, K.A., Elias, S.C., Halstead, F.D., Goodman, A.L., Edwards, N.J., Reyes-Sandoval, A., Bird, P., et al. (2012). Clinical assessment of a recombinant simian adenovirus ChAd63: a potent new vaccine vector. J. Infect. Dis. *205*, 772–781.

9. Keefer, M.C., Gilmour, J., Hayes, P., Gill, D., Kopycinski, J., Cheeseman, H., Cashin-Cox, M., Naarding, M., Clark, L., and Fernandez, N. (2012). A Phase I Double Blind, Placebo-Controlled, Randomized Study of a Multigenic HIV-1 Adenovirus Subtype 35 Vector Vaccine in Healthy Uninfected Adults. PLoS One *7*, e41936.

10. Ouédraogo, A., Tiono, A.B., Kargougou, D., Yaro, J.B., Ouédraogo, E., Kaboré, Y., Kangoye, D., Bougouma, E.C., Gansane, A., Henri, N., et al. (2013). A phase 1b randomized, controlled, double-blinded dosage-escalation trial to evaluate the safety, reactogenicity and immunogenicity of an adenovirus type 35 based circumsporozoite malaria vaccine in Burkinabe healthy adults 18 to 45 years of age. PLoS One *8*, e78679.

11. Williams, K., Bastian, A.R., Feldman, R.A., Omoruyi, E., de Paepe, E., Hendriks, J., van Zeeburg, H., Godeaux, O., Langedijk, J.P.M., Schuitemaker, H., et al. (2020). Phase 1 safety and immunogenicity study of a respiratory syncytial virus vaccine with an adenovirus 26 vector encoding prefusion F (Ad26. RSV. preF) in adults aged≥ 60 years. J. Infect. Dis. *222*, 979–988.

12. Stephenson, K.E., Le Gars, M., Sadoff, J., de Groot, A.M., Heerwegh, D., Truyers, C., Atyeo, C., Loos, C., Chandrashekar, A., McMahan, K., et al. (2021). Immunogenicity of the Ad26. COV2. S Vaccine for COVID-19. JAMA *325*, 1535–1544.

13. Sadoff, J., Gray, G., Vandebosch, A., Cárdenas, V., Shukarev, G., Grinsztejn, B., Goepfert, P.A., Truyers, C., Fennema, H., Spiessens, B., et al. (2021). Safety and efficacy of single-dose Ad26. COV2. S vaccine against Covid-19. N. Engl. J. Med. *384*, 2187–2201.

14. Folegatti, P.M., Ewer, K.J., Aley, P.K., Angus, B., Becker, S., Belij-Rammerstorfer, S., Bellamy, D., Bibi, S., Bittaye, M., Clutterbuck, E.A., et al. (2020). Safety and immunogenicity of the ChAdOx1 nCoV-19 vaccine against SARS-CoV-2: a preliminary report of a phase 1/2, single-blind, randomised controlled trial. Lancet *396*, 467–478.

15. Voysey, M., Clemens, S.A.C., Madhi, S.A., Weckx, L.Y., Folegatti, P.M., Aley, P.K., Angus, B., Baillie, V.L., Barnabas, S.L., Bhorat, Q.E., et al. (2021). Safety and efficacy of the ChAdOx1 nCoV-19 vaccine (AZD1222) against SARS-CoV-2: an interim analysis of four randomised controlled trials in Brazil, South Africa, and the UK. Lancet *397*, 99–111.

16. Ewer, K.J., Barrett, J.R., Belij-Rammerstorfer, S., Sharpe, H., Makinson, R., Morter, R., Flaxman, A., Wright, D., Bellamy, D., Bittaye, M., et al. (2021). T cell and antibody responses induced by a single dose of ChAdOx1 nCoV-19 (AZD1222) vaccine in a phase 1/2 clinical trial. Nat. Med. *27*, 270–278.

17. Hassan, A.O., Kafai, N.M., Dmitriev, I.P., Fox, J.M., Smith, B.K., Harvey, I.B., Chen, R.E., Winkler, E.S., Wessel, A.W., Case, J.B., et al. (2020). A Single-Dose Intranasal ChAd Vaccine Protects Upper and Lower Respiratory Tracts against SARS-CoV-2. Cell *183*, 169–184.e13. https://doi.org/10.1016/j.cell.2020.08.026.

18. Colloca, S., Barnes, E., Folgori, A., Ammendola, V., Capone, S., Cirillo, A., Siani, L., Naddeo, M., Grazioli, F., Esposito, M.L., et al. (2012). Vaccine vectors derived from a large collection of simian adenoviruses induce potent cellular immunity across multiple species. Sci. Transl. Med. *4*, 115ra2.

19. Bliss, C.M., Parsons, A.J., Nachbagauer, R., Hamilton, J.R., Cappuccini, F., Ulaszewska, M., Webber, J.P., Clayton, A., Hill, A.V.S., and Coughlan, L. (2020). Targeting antigen to the surface of EVs improves the in vivo immunogenicity of human and non-human adenoviral vaccines in mice. Mol. Ther. Methods Clin. Dev. *16*, 108–125.

20. Coughlan, L. (2020). Factors which contribute to the immunogenicity of non-replicating adenoviral vectored vaccines. Front. Immunol. *11*, 909.

21. Zhu, F.-C., Guan, X.-H., Li, Y.-H., Huang, J.-Y., Jiang, T., Hou, L.-H., Li, J.-X., Yang, B.-F., Wang, L., Wang, W.-J., et al. (2020). Immunogenicity and safety of a recombinant adenovirus type-5-vectored COVID-19 vaccine in healthy adults aged 18 years or older: a randomised, double-blind, placebo-controlled, phase 2 trial. Lancet *396*, 479–488.

22. Buchbinder, S.P., Mehrotra, D.V., Duerr, A., Fitzgerald, D.W., Mogg, R., Li, D., Gilbert, P.B., Lama, J.R., Marmor, M., Del Rio, C., et al. (2008). Efficacy assessment of a cell-mediated immunity HIV-1 vaccine (the Step Study): a double-blind, randomised, placebo-controlled, test-of-concept trial. Lancet *372*, 1881–1893.

23. Halperin, S.A., Ye, L., MacKinnon-Cameron, D., Smith, B., Cahn, P.E., Ruiz-Palacios, G.M., Ikram, A., Lanas, F., Lourdes Guerrero, M., Muñoz Navarro, S.R., et al. (2022). Final efficacy analysis, interim safety analysis, and immunogenicity of a single dose of recombinant novel coronavirus vaccine (adenovirus type 5 vector) in adults 18 years and older: an international, multicentre, randomised, double-blinded, placebo-controlled phase 3 trial. Lancet *399*, 237–248.

24. Logunov, D.Y., Dolzhikova, I.V., Shcheblyakov, D.V., Tukhvatulin, A.I., Zubkova, O.V., Dzharullaeva, A.S., Kovyrshina, A.V., Lubenets, N.L., Grousova, D.M., Erokhova, A.S., et al. (2021). Safety and efficacy of an rAd26 and rAd5 vector-based heterologous prime-boost COVID-19 vaccine: an interim analysis of a randomised controlled phase 3 trial in Russia. Lancet *397*, 671–681.

25. Gabitzsch, E.S., Tsang, K.Y., Palena, C., David, J.M., Fantini, M., Kwilas, A., Rice, A.E., Latchman, Y., Hodge, J.W., Gulley, J.L., et al. (2015). The generation and analyses of a novel combination of recombinant adenovirus vaccines targeting three tumor antigens as an immunotherapeutic. Oncotarget *6*, 31344–31359.

26. Milligan, I.D., Gibani, M.M., Sewell, R., Clutterbuck, E.A., Campbell, D., Plested, E., Nuthall, E., Voysey, M., Silva-Reyes, L., McElrath, M.J., et al. (2016). Safety and Immunogenicity of Novel Adenovirus Type 26- and Modified Vaccinia Ankara-Vectored Ebola Vaccines: A Randomized Clinical Trial. JAMA *315*, 1610–1623. https://doi.org/10.1001/jama.2016.4218.

27. Bergelson, J.M., Cunningham, J.A., Droguett, G., Kurt-Jones, E.A., Krithivas, A., Hong, J.S., Horwitz, M.S., Crowell, R.L., and Finberg, R.W. (1997). Isolation of a common receptor for Coxsackie B viruses and adenoviruses 2 and 5. Science *275*, 1320–1323.

28. Cohen, C.J., Shieh, J.T., Pickles, R.J., Okegawa, T., Hsieh, J.-T., and Bergelson, J.M. (2001). The coxsackievirus and adenovirus receptor is a transmembrane component of the tight junction. Proc. Natl. Acad. Sci. USA *98*, 15191–15196.

29. Carlisle, R.C., Di, Y., Cerny, A.M., Sonnen, A.F.-P., Sim, R.B., Green, N.K., Subr, V., Ulbrich, K., Gilbert, R.J.C., Fisher, K.D., et al. (2009). Human erythrocytes bind and inactivate type 5 adenovirus by presenting Coxsackie virus-adenovirus receptor and complement receptor 1. Blood J. Am. Soc. Hematol. *113*, 1909–1918.

30. Baker, A.T., Boyd, R.J., Sarkar, D., Teijeira-Crespo, A., Chan, C.K., Bates, E., Waraich, K., Vant, J., Wilson, E., Truong, C.D., et al. (2021). ChAdOx1 interacts with CAR and PF4 with implications for thrombosis with thrombocytopenia syndrome. Sci. Adv. *7*, eabl8213. https://doi.org/10.1126/sciadv.abl8213.

31. Wickham, T.J., Filardo, E.J., Cheresh, D.A., and Nemerow, G.R. (1994). Integrin alpha v beta 5 selectively promotes adenovirus mediated cell membrane permeabilization. J. Cell Biol. *127*, 257–264.

32. Coughlan, L., Alba, R., Parker, A.L., Bradshaw, A.C., McNeish, I.A., Nicklin, S.A., and Baker, A.H. (2010). Tropism-modification strategies for targeted gene delivery using adenoviral vectors. Viruses *2*, 2290–2355.

33. Uusi-Kerttula, H., Hulin-Curtis, S., Davies, J., and Parker, A.L. (2015). Oncolytic adenovirus: strategies and insights for vector design and immuno-oncolytic applications. Viruses *7*, 6009–6042.

34. Baker, A.T., Davies, J.A., Bates, E.A., Moses, E., Mundy, R.M., Marlow, G., Cole, D.K., Bliss, C.M., Rizkallah, P.J., and Parker, A.L. (2021). The fiber knob protein

of human adenovirus type 49 mediates highly efficient and promiscuous infection of cancer cell lines using a novel cell entry mechanism. J. Virol. 95, e018499-20.

35. Segerman, A., Arnberg, N., Erikson, A., Lindman, K., and Wadell, G. (2003). There are two different species B adenovirus receptors: sBAR, common to species B1 and B2 adenoviruses, and sB2AR, exclusively used by species B2 adenoviruses. J. Virol. 77, 1157–1162.

36. Arnberg, N. (2009). Adenovirus receptors: implications for tropism, treatment and targeting. Rev. Med. Virol. 19, 165–178.

37. Vogels, R., Zuijdgeest, D., van Rijnsoever, R., Hartkoorn, E., Damen, I., de Béthune, M.-P., Kostense, S., Penders, G., Helmus, N., Koudstaal, W., et al. (2003). Replication-deficient human adenovirus type 35 vectors for gene transfer and vaccination: efficient human cell infection and bypass of preexisting adenovirus immunity. J. Virol. 77, 8263–8271. https://doi.org/10.1128/jvi.77.15.8263-8271.2003.

38. Lemckert, A.A.C., Grimbergen, J., Smits, S., Hartkoorn, E., Holterman, L., Berkhout, B., Barouch, D.H., Vogels, R., Quax, P., Goudsmit, J., and Havenga, M.J.E. (2006). Generation of a novel replication-incompetent adenoviral vector derived from human adenovirus type 49: manufacture on PER.C6 cells, tropism and immunogenicity. J. Gen. Virol. 87, 2891–2899. https://doi.org/10.1099/vir.0.82079-0.

39. Handy, C.E., and Antonarakis, E.S. (2018). Sipuleucel-T for the treatment of prostate cancer: novel insights and future directions. Future Oncol. 14, 907–917. https://doi.org/10.2217/fon-2017-0531.

40. Shortman, K., Lahoud, M.H., and Caminschi, I. (2009). Improving vaccines by targeting antigens to dendritic cells. Exp. Mol. Med. 41, 61–66. https://doi.org/10.3858/emm.2009.41.2.008.

41. Zanna, M.Y., Yasmin, A.R., Omar, A.R., Arshad, S.S., Mariatulqabtiah, A.R., Nur-Fazila, S.H., and Mahiza, M.I.N. (2021). Review of Dendritic Cells, Their Role in Clinical Immunology, and Distribution in Various Animal Species. Int. J. Mol. Sci. 22, 8044. https://doi.org/10.3390/ijms22158044.

42. Loré, K., Adams, W.C., Havenga, M.J.E., Precopio, M.L., Holterman, L., Goudsmit, J., and Koup, R.A. (2007). Myeloid and plasmacytoid dendritic cells are susceptible to recombinant adenovirus vectors and stimulate polyfunctional memory T cell responses. J. Immunol. Baltim. Md 1950 179, 1721–1729. https://doi.org/10.4049/jimmunol.179.3.1721.

43. Adams, W.C., Bond, E., Havenga, M.J.E., Holterman, L., Goudsmit, J., Karlsson Hedestam, G.B., Koup, R.A., and Loré, K. (2009). Adenovirus serotype 5 infects human dendritic cells via a coxsackievirus-adenovirus receptor-independent receptor pathway mediated by lactoferrin and DC-SIGN. J. Gen. Virol. 90, 1600–1610. https://doi.org/10.1099/vir.0.008342-0.

44. Cheng, C., Gall, J.G.D., Kong, W.p., Sheets, R.L., Gomez, P.L., King, C.R., and Nabel, G.J. (2007). Mechanism of ad5 vaccine immunity and toxicity: fiber shaft targeting of dendritic cells. PLoS Pathog. 3, e25. https://doi.org/10.1371/journal.ppat.0030025.

45. Dakin, R.S., Parker, A.L., Delles, C., Nicklin, S.A., and Baker, A.H. (2015). Efficient transduction of primary vascular cells by the rare adenovirus serotype 49 vector. Hum. Gene Ther. 26, 312–319. https://doi.org/10.1089/hum.2015.019.

46. Motwani, J.G., and Topol, E.J. (1998). Aortocoronary saphenous vein graft disease: pathogenesis, predisposition, and prevention. Circulation 97, 916–931. https://doi.org/10.1161/01.cir.97.9.916.

47. Parker, A.L., White, K.M., Lavery, C.A., Custers, J., Waddington, S.N., and Baker, A.H. (2013). Pseudotyping the adenovirus serotype 5 capsid with both the fibre and penton of serotype 35 enhances vascular smooth muscle cell transduction. Gene Ther. 20, 1158–1164. https://doi.org/10.1038/gt.2013.44.

48. Nicklin, S.A., and Baker, A.H. (2003). Development of targeted viral vectors for cardiovascular gene therapy. Genet. Eng. 25, 15–49. https://doi.org/10.1007/978-1-4615-0073-5_2.

49. Stern, P.L., and Harrop, R. (2017). 5T4 oncofoetal antigen: an attractive target for immune intervention in cancer. Cancer Immunol. Immunother. 66, 415–426. https://doi.org/10.1007/s00262-016-1917-3.

50. Graham, S.P., McLean, R.K., Spencer, A.J., Belij-Rammerstorfer, S., Wright, D., Ulaszewska, M., Edwards, J.C., Hayes, J.W.P., Martini, V., Thakur, N., et al. (2020). Evaluation of the immunogenicity of prime-boost vaccination with the replication-deficient viral vectored COVID-19 vaccine candidate ChAdOx1 nCoV-19. NPJ Vaccines 5, 69. https://doi.org/10.1038/s41541-020-00221-3.

51. Bliss, C.M., Freyn, A.W., Caniels, T.G., Leyva-Grado, V.H., Nachbagauer, R., Sun, W., Tan, G.S., Gillespie, V.L., McMahon, M., Krammer, F., et al. (2022). A single-shot adenoviral vaccine provides hemagglutinin stalk-mediated protection against heterosubtypic influenza challenge in mice. Mol. Ther. 30, 2024–2047. https://doi.org/10.1016/j.ymthe.2022.01.011.

52. Cox, F., van der Fits, L., Abbink, P., Larocca, R.A., van Huizen, E., Saeland, E., Verhagen, J., Peterson, R., Tolboom, J., Kaufmann, B., et al. (2018). Adenoviral vector type 26 encoding Zika virus (ZIKV) M-Env antigen induces humoral and cellular immune responses and protects mice and nonhuman primates against ZIKV challenge. PLoS One 13, e0202820. https://doi.org/10.1371/journal.pone.0202820.

53. Liu, J., Ewald, B.A., Lynch, D.M., Denholtz, M., Abbink, P., Lemckert, A.A.C., Carville, A., Mansfield, K.G., Havenga, M.J., Goudsmit, J., and Barouch, D.H. (2008). Magnitude and phenotype of cellular immune responses elicited by recombinant adenovirus vectors and heterologous prime-boost regimens in rhesus monkeys. J. Virol. 82, 4844–4852. https://doi.org/10.1128/JVI.02616-07.

54. Boyd, A., Almeida, J.R., Darrah, P.A., Sauce, D., Seder, R.A., Appay, V., Gorochov, G., and Larsen, M. (2015). Correction: Pathogen-Specific T Cell Polyfunctionality Is a Correlate of T Cell Efficacy and Immune Protection. PLoS One 10, e0138395. https://doi.org/10.1371/journal.pone.0138395.

55. Darrah, P.A., Patel, D.T., De Luca, P.M., Lindsay, R.W.B., Davey, D.F., Flynn, B.J., Hoff, S.T., Andersen, P., Reed, S.G., Morris, S.L., et al. (2007). Multifunctional TH1 cells define a correlate of vaccine-mediated protection against Leishmania major. Nat. Med. 13, 843–850. https://doi.org/10.1038/nm1592.

56. Casazza, J.P., Betts, M.R., Price, D.A., Precopio, M.L., Ruff, L.E., Brenchley, J.M., Hill, B.J., Roederer, M., Douek, D.C., and Koup, R.A. (2006). Acquisition of direct antiviral effector functions by CMV-specific CD4+ T lymphocytes with cellular maturation. J. Exp. Med. 203, 2865–2877. https://doi.org/10.1084/jem.20052246.

57. Ding, Z.-C., Huang, L., Blazar, B.R., Yagita, H., Mellor, A.L., Munn, D.H., and Zhou, G. (2012). Polyfunctional $CD4^{+}$ T cells are essential for eradicating advanced B-cell lymphoma after chemotherapy. Blood 120, 2229–2239. https://doi.org/10.1182/blood-2011-12-398321.

58. Ewer, K.J., O'Hara, G.A., Duncan, C.J.A., Collins, K.A., Sheehy, S.H., Reyes-Sandoval, A., Goodman, A.L., Edwards, N.J., Elias, S.C., Halstead, F.D., et al. (2013). Protective CD8+ T-cell immunity to human malaria induced by chimpanzee adenovirus-MVA immunisation. Nat. Commun. 4, 2836. https://doi.org/10.1038/ncomms3836.

59. Betts, M.R., Nason, M.C., West, S.M., De Rosa, S.C., Migueles, S.A., Abraham, J., Lederman, M.M., Benito, J.M., Goepfert, P.A., Connors, M., et al. (2006). HIV nonprogressors preferentially maintain highly functional HIV-specific CD8+ T cells. Blood 107, 4781–4789. https://doi.org/10.1182/blood-2005-12-4818.

60. Channappanavar, R., Fett, C., Zhao, J., Meyerholz, D.K., and Perlman, S. (2014). Virus-specific memory CD8 T cells provide substantial protection from lethal severe acute respiratory syndrome coronavirus infection. J. Virol. 88, 11034–11044. https://doi.org/10.1128/JVI.01505-14.

61. Imai, N., Tawara, I., Yamane, M., Muraoka, D., Shiku, H., and Ikeda, H. (2020). CD4+ T cells support polyfunctionality of cytotoxic CD8+ T cells with memory potential in immunological control of tumor. Cancer Sci. 111, 1958–1968. https://doi.org/10.1111/cas.14420.

62. Precopio, M.L., Betts, M.R., Parrino, J., Price, D.A., Gostick, E., Ambrozak, D.R., Asher, T.E., Douek, D.C., Harari, A., Pantaleo, G., et al. (2007). Immunization with vaccinia virus induces polyfunctional and phenotypically distinctive CD8(+) T cell responses. J. Exp. Med. 204, 1405–1416. https://doi.org/10.1084/jem.20062363.

63. Sadoff, J., Le Gars, M., Shukarev, G., Heerwegh, D., Truyers, C., de Groot, A.M., Stoop, J., Tete, S., Van Damme, W., Leroux-Roels, I., et al. (2021). Interim Results of a Phase 1-2a Trial of Ad26.COV2.S Covid-19 Vaccine. N. Engl. J. Med. 384, 1824–1835. https://doi.org/10.1056/NEJMoa2034201.

64. Tapia, M.D., Sow, S.O., Lyke, K.E., Haidara, F.C., Diallo, F., Doumbia, M., Traore, A., Coulibaly, F., Kodio, M., Onwuchekwa, U., et al. (2016). Use of ChAd3-EBO-Z Ebola virus vaccine in Malian and US adults, and boosting of Malian adults with MVA-BN-Filo: a phase 1, single-blind, randomised trial, a phase 1b, open-label and double-blind, dose-escalation trial, and a nested, randomised, double-blind, placebo-controlled trial. Lancet Infect. Dis. 16, 31–42. https://doi.org/10.1016/S1473-3099(15)00362-X.

65. Ledgerwood, J.E., Costner, P., Desai, N., Holman, L., Enama, M.E., Yamshchikov, G., Mulangu, S., Hu, Z., Andrews, C.A., Sheets, R.A., et al. (2010). A replication defective recombinant Ad5 vaccine expressing Ebola virus GP is safe and immunogenic in healthy adults. Vaccine *29*, 304–313. https://doi.org/10.1016/j.vaccine.2010.10.037.

66. Hofmeyer, K.A., Bianchi, K.M., and Wolfe, D.N. (2022). Utilization of Viral Vector Vaccines in Preparing for Future Pandemics. Vaccines *10*, 436. https://doi.org/10.3390/vaccines10030436.

67. Bos, R., Rutten, L., van der Lubbe, J.E.M., Bakkers, M.J.G., Hardenberg, G., Wegmann, F., Zuijdgeest, D., de Wilde, A.H., Koornneef, A., Verwilligen, A., et al. (2020). Ad26 vector-based COVID-19 vaccine encoding a prefusion-stabilized SARS-CoV-2 Spike immunogen induces potent humoral and cellular immune responses. NPJ Vaccines *5*, 91. https://doi.org/10.1038/s41541-020-00243-x.

68. Cheng, C., Gall, J.G.D., Nason, M., King, C.R., Koup, R.A., Roederer, M., McElrath, M.J., Morgan, C.A., Churchyard, G., Baden, L.R., et al. (2010). Gary J Nabel Differential specificity and immunogenicity of adenovirus type 5 neutralizing antibodies elicited by natural infection or immunization. J. Virol. *84*, 630–638. https://doi.org/10.1128/JVI.00866-09.

69. Luo, K., Gordy, J.T., Zavala, F., and Markham, R.B. (2021). A chemokine-fusion vaccine targeting immature dendritic cells elicits elevated antibody responses to malaria sporozoites in infant macaques. Sci. Rep. *11*, 1220. https://doi.org/10.1038/s41598-020-79427-3.

70. George, R., Ma, A., Motyka, B., Shi, Y.E., Liu, Q., and Griebel, P. (2020). A dendritic cell-targeted chimeric hepatitis B virus immunotherapeutic vaccine induces both cellular and humoral immune responses in vivo. Hum. Vaccin. Immunother. *16*, 779–792. https://doi.org/10.1080/21645515.2019.1689081.

71. Tanyi, J.L., Bobisse, S., Ophir, E., Tuyaerts, S., Roberti, A., Genolet, R., Baumgartner, P., Stevenson, B.J., Iseli, C., Dangaj, D., et al. (2018). Personalized cancer vaccine effectively mobilizes antitumor T cell immunity in ovarian cancer. Sci. Transl. Med. *10*, eaao5931. https://doi.org/10.1126/scitranslmed.aao5931.

72. Kastenmüller, W., Kastenmüller, K., Kurts, C., and Seder, R.A. (2014). Dendritic cell-targeted vaccines–hope or hype? Nat. Rev. Immunol. *14*, 705–711. https://doi.org/10.1038/nri3727.

73. Tada, F., Abe, M., Hirooka, M., Ikeda, Y., Hiasa, Y., Lee, Y., Jung, N.-C., Lee, W.-B., Lee, H.-S., Bae, Y.-S., and Onji, M. (2012). Phase I/II study of immunotherapy using tumor antigen-pulsed dendritic cells in patients with hepatocellular carcinoma. Int. J. Oncol. *41*, 1601–1609. https://doi.org/10.3892/ijo.2012.1626.

74. Schuler, P.J., Harasymczuk, M., Visus, C., Deleo, A., Trivedi, S., Lei, Y., Argiris, A., Gooding, W., Butterfield, L.H., Whiteside, T.L., and Ferris, R.L. (2014). Phase I dendritic cell p53 peptide vaccine for head and neck cancer. Clin. Cancer Res. *20*, 2433–2444. https://doi.org/10.1158/1078-0432.CCR-13-2617.

75. Jonuleit, H., Giesecke-Tuettenberg, A., Tüting, T., Thurner-Schuler, B., Stuge, T.B., Paragnik, L., Kandemir, A., Lee, P.P., Schuler, G., Knop, J., and Enk, A.H. (2001). A comparison of two types of dendritic cell as adjuvants for the induction of melanoma-specific T-cell responses in humans following intranodal injection. Int. J. Cancer *93*, 243–251. https://doi.org/10.1002/ijc.1323.

76. Oshita, C., Takikawa, M., Kume, A., Miyata, H., Ashizawa, T., Iizuka, A., Kiyohara, Y., Yoshikawa, S., Tanosaki, R., Yamazaki, N., et al. (2012). Dendritic cell-based vaccination in metastatic melanoma patients: phase II clinical trial. Oncol. Rep. *28*, 1131–1138. https://doi.org/10.3892/or.2012.1956.

77. Chang, A.E., Redman, B.G., Whitfield, J.R., Nickoloff, B.J., Braun, T.M., Lee, P.P., Geiger, J.D., and Mulé, J.J. (2002). A phase I trial of tumor lysate-pulsed dendritic cells in the treatment of advanced cancer. Clin. Cancer Res. 8, 1021–1032.

78. Hunyadi, J., András, C., Szabó, I., Szántó, J., Szluha, K., Sipka, S., Kovács, P., Kiss, A., Szegedi, G., Altorjay, I., et al. (2014). Autologous dendritic cell based adoptive immunotherapy of patients with colorectal cancer-A phase I-II study. Pathol. Oncol. Res. *20*, 357–365. https://doi.org/10.1007/s12253-013-9704-3.

79. Osada, T., Nagaoka, K., Takahara, M., Yang, X.Y., Liu, C.-X., Guo, H., Roy Choudhury, K., Hobeika, A., Hartman, Z., Morse, M.A., and Lyerly, H.K. (2015). Precision cancer immunotherapy: optimizing dendritic cell-based strategies to induce tumor antigen-specific T-cell responses against individual patient tumors. J. Immunother. *38*, 155–164. https://doi.org/10.1097/CJI.0000000000000075.

80. Aarntzen, E.H.J.G., Schreibelt, G., Bol, K., Lesterhuis, W.J., Croockewit, A.J., de Wilt, J.H.W., van Rossum, M.M., Blokx, W.A.M., Jacobs, J.F.M., Duiveman-de Boer, T., et al. (2012). Vaccination with mRNA-electroporated dendritic cells induces robust tumor antigen-specific CD4+ and CD8+ T cells responses in stage III and IV melanoma patients. Clin. Cancer Res. *18*, 5460–5470. https://doi.org/10.1158/1078-0432.CCR-11-3368.

81. Nair, S.K., Morse, M., Boczkowski, D., Cumming, R.I., Vasovic, L., Gilboa, E., and Lyerly, H.K. (2002). Induction of tumor-specific cytotoxic T lymphocytes in cancer patients by autologous tumor RNA-transfected dendritic cells. Ann. Surg. *235*, 540–549. https://doi.org/10.1097/00000658-200204000-00013.

82. Vik-Mo, E.O., Nyakas, M., Mikkelsen, B.V., Moe, M.C., Due-Tønnesen, P., Suso, E.M.I., Sæbøe-Larssen, S., Sandberg, C., Brinchmann, J.E., Helseth, E., et al. (2013). Therapeutic vaccination against autologous cancer stem cells with mRNA-transfected dendritic cells in patients with glioblastoma. Cancer Immunol. Immunother. *62*, 1499–1509. https://doi.org/10.1007/s00262-013-1453-3.

83. Morse, M.A., Chapman, R., Powderly, J., Blackwell, K., Keler, T., Green, J., Riggs, R., He, L.-Z., Ramakrishna, V., Vitale, L., et al. (2011). Phase I study utilizing a novel antigen-presenting cell-targeted vaccine with Toll-like receptor stimulation to induce immunity to self-antigens in cancer patients. Clin. Cancer Res. *17*, 4844–4853. https://doi.org/10.1158/1078-0432.CCR-11-0891.

84. Sharma, P.K., Dmitriev, I.P., Kashentseva, E.A., Raes, G., Li, L., Kim, S.W., Lu, Z.-H., Arbeit, J.M., Fleming, T.P., Kaliberov, S.A., et al. (2018). Development of an adenovirus vector vaccine platform for targeting dendritic cells. Cancer Gene Ther. *25*, 27–38. https://doi.org/10.1038/s41417-017-0002-1.

85. Belousova, N., Korokhov, N., Krendelshchikova, V., Simonenko, V., Mikheeva, G., Triozzi, P.L., Aldrich, W.A., Banerjee, P.T., Gillies, S.D., Curiel, D.T., and Krasnykh, V. (2003). Genetically targeted adenovirus vector directed to CD40-expressing cells. J. Virol. *77*, 11367–11377. https://doi.org/10.1128/jvi.77.21.11367-11377.2003.

86. Hangalapura, B.N., Timares, L., Oosterhoff, D., Scheper, R.J., Curiel, D.T., and de Gruijl, T.D. (2012). CD40-targeted adenoviral cancer vaccines: the long and winding road to the clinic. J. Gene Med. *14*, 416–427. https://doi.org/10.1002/jgm.1648.

87. Shimamura, M., Nakagami, H., Sanada, F., and Morishita, R. (2020). Progress of Gene Therapy in Cardiovascular Disease. Hypertens. Dallas Tex, 1038–1044. https://doi.org/10.1161/HYPERTENSIONAHA.120.14478.

88. Ylä-Herttuala, S., and Baker, A.H. (2017). Cardiovascular Gene Therapy: Past, Present, and Future. Mol. Ther. *25*, 1095–1106. https://doi.org/10.1016/j.ymthe.2017.03.027.

89. Conte, M.S., Bandyk, D.F., Clowes, A.W., Moneta, G.L., Seely, L., Lorenz, T.J., Namini, H., Hamdan, A.D., Roddy, S.P., Belkin, M., et al. (2006). Results of PREVENT III: a multicenter, randomized trial of edifoligide for the prevention of vein graft failure in lower extremity bypass surgery. J. Vasc. Surg. *43*, 742–751. , discussion 751. https://doi.org/10.1016/j.jvs.2005.12.058.

90. Alexander, J.H., Hafley, G., Harrington, R.A., Peterson, E.D., Ferguson, T.B., Lorenz, T.J., Goyal, A., Gibson, M., Mack, M.J., Gennevois, D., et al. (2005). Efficacy and safety of edifoligide, an E2F transcription factor decoy, for prevention of vein graft failure following coronary artery bypass graft surgery: PREVENT IV: a randomized controlled trial. JAMA *294*, 2446–2454. https://doi.org/10.1001/jama.294.19.2446.

91. Kritz, A.B., Yu, J., Wright, P.L., Wan, S., George, S.J., Halliday, C., Kang, N., Sessa, W.C., and Baker, A.H. (2008). In Vivo Modulation of Nogo-B Attenuates Neointima Formation. Mol. Ther. *16*, 1798–1804. https://doi.org/10.1038/mt.2008.188.

92. Stanton, R.J., McSharry, B.P., Armstrong, M., Tomasec, P., and Wilkinson, G.W.G. (2008). Re-engineering adenovirus vector systems to enable high-throughput analyses of gene function. Biotechniques *45*, 659–668. https://doi.org/10.2144/000112993.

93. Stanton, R.J. (2008). pAL1141. https://adz.cf.ac.uk/sites/default/files/pAL1141.gb.

94. Recombineering using SacB cassette: https://www.cardiff.ac.uk/__data/assets/pdf_file/0017/517103/Cloning-genes-into-the-AdZ-vectors-and-making-virus.pdf.

95. Recombineering using rpsL cassette: https://www.cardiff.ac.uk/__data/assets/pdf_file/0016/1026052/General-Recombineering.pdf.

96. (2006). Fiber [Human adenovirus 49], GenBank: ABD52400.1. https://www.ncbi.nlm.nih.gov/protein/88810187.

97. Baker, A.T., Mundy, R.M., Davies, J.A., Rizkallah, P.J., and Parker, A.L. (2019). Human adenovirus type 26 uses sialic acid-bearing glycans as a primary cell entry receptor. Sci. Adv. *5*, eaax3567. https://doi.org/10.1126/sciadv.aax3567.

98. Baker, A.T., Greenshields-Watson, A., Coughlan, L., Davies, J.A., Uusi-Kerttula, H., Cole, D.K., Rizkallah, P.J., and Parker, A.L. (2019). Diversity within the adenovirus fiber knob hypervariable loops influences primary receptor interactions. Nat. Commun. *10*, 741. https://doi.org/10.1038/s41467-019-08599-y.

99. Uusi-Kerttula, H., Davies, J., Coughlan, L., Hulin-Curtis, S., Jones, R., Hanna, L., Chester, J.D., and Parker, A.L. (2016). Pseudotyped αvβ6 integrin-targeted adenovirus vectors for ovarian cancer therapies. Oncotarget *7*, 27926–27937. https://doi.org/10.18632/oncotarget.8545.

100. Uusi-Kerttula, H., Legut, M., Davies, J., Jones, R., Hudson, E., Hanna, L., Stanton, R.J., Chester, J.D., and Parker, A.L. (2015). Incorporation of Peptides Targeting EGFR and FGFR1 into the Adenoviral Fiber Knob Domain and Their Evaluation as Targeted Cancer Therapies. Hum. Gene Ther. *26*, 320–329. https://doi.org/10.1089/hum.2015.015.

101. Dwivedi, A., Sala-Newby, G.B., and George, S.J. (2008). Regulation of cell–matrix contacts and β-catenin signaling in VSMC by integrin-linked kinase: implications for intimal thickening. Basic Res. Cardiol. *103*, 244–256. https://doi.org/10.1007/s00395-007-0693-9.

102. Kilkenny, C., Browne, W.J., Cuthi, I., Emerson, M., and Altman, D.G. (2012). Improving bioscience research reporting: the ARRIVE guidelines for reporting animal research. Vet. Clin. Pathol. *41*, 27–31. https://doi.org/10.1111/j.1939-165X.2012.00418.x.

OMTM, Volume *32*

Supplemental information

A pseudotyped adenovirus serotype 5 vector with serotype 49 fiber knob is an effective vector for vaccine and gene therapy applications

Carly M. Bliss, Sarah L. Hulin-Curtis, Marta Williams, Mahulena Marušková, James A. Davies, Evelina Statkute, Alexander T. Baker, Louise Stack, Lucas Kerstetter, Lauren E. Kerr-Jones, Kate F. Milward, Gabrielle Russell, Sarah J. George, Luned M. Badder, Richard J. Stanton, Lynda Coughlan, Ian R. Humphreys, and Alan L. Parker

Table S1 – Flow cytometry antibody panel used in Figure 3

Marker	Staining Cocktail	Fluorochrome	Antibody Clone	Dilution	Stock Concentration	Source
**CD3**	Surface	Pacific Blue	17A2	1:100	0.5mg/mL	Biolegend
**CD4**	Surface	BV510	RM4-5	1:150	0.2mg/mL	Biolegend
**CD8**	Surface	AlexaFluor700	53-6.7	1:50	0.2mg/mL	eBioscience
**LIVE/ DEAD**	Surface	Near Infrared	N/A	1:1000	N/A	ThermoFisher
**IL-2**	Intracellular	PE-Cy7	JES6-5H4	1:250	0.2mg/mL	eBioscience
**IFN-γ**	Intracellular	eFluor660	XMG1.2	1:500	0.2mg/mL	eBioscience
**TNFα**	Intracellular	AlexaFluor488	MP6-XT22	1:2000	0.5mg/mL	eBioscience
**CD40L**	Intracellular	PerCP eFluor710	MR1	1:150	0.2mg/mL	ThermoFisher

Table S2 – Flow cytometry antibody panel used in Figure 5

Marker	Staining Cocktail	Fluorochrome	Antibody Clone	Dilution	Stock Concentration	Source
**CD4**	Surface	APC	GK1.5	1:100	0.2mg/mL	Biolegend
**CD8**	Surface	PE-Cy7	53-6.7	1:50	0.2mg/mL	Biolegend
**LIVE/ DEAD**	Surface	Zombie Aqua	N/A	1:500	N/A	Biolegend
**IFN-γ**	Intracellular	FITC	XMG1.2	1:100	0.5mg/mL	Biolegend
**TNFα**	Intracellular	PE	MP6-XT22	1:100	0.2mg/mL	Biolegend

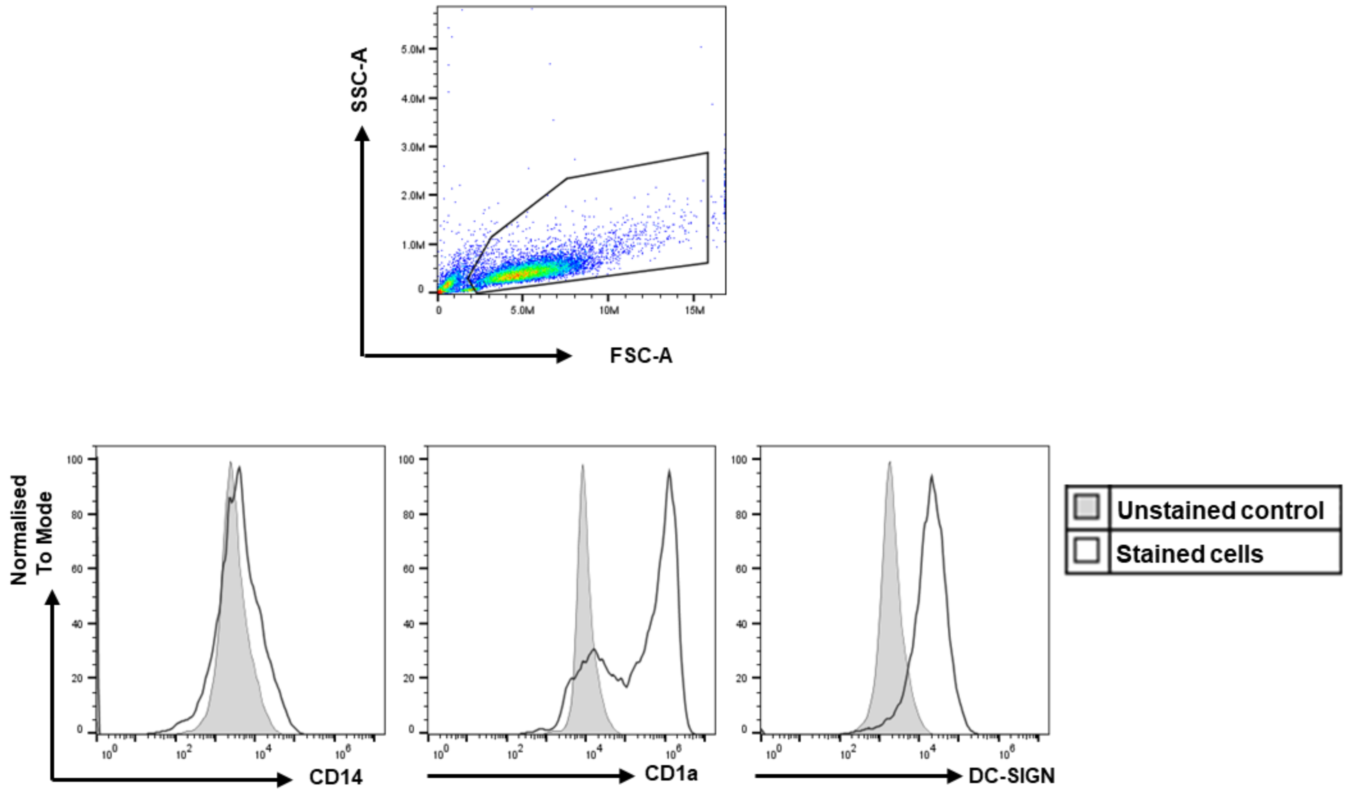

Figure S1 – Validation of human monocyte-derived dendritic cells using surface marker expression

Following *in vitro* differentiation from $CD14^+$ enriched PBMC, dendritic cell phenotype was confirmed by flow cytometry using $CD14^-$ $CD1a^+$ DC-SIGN$^+$ validation.

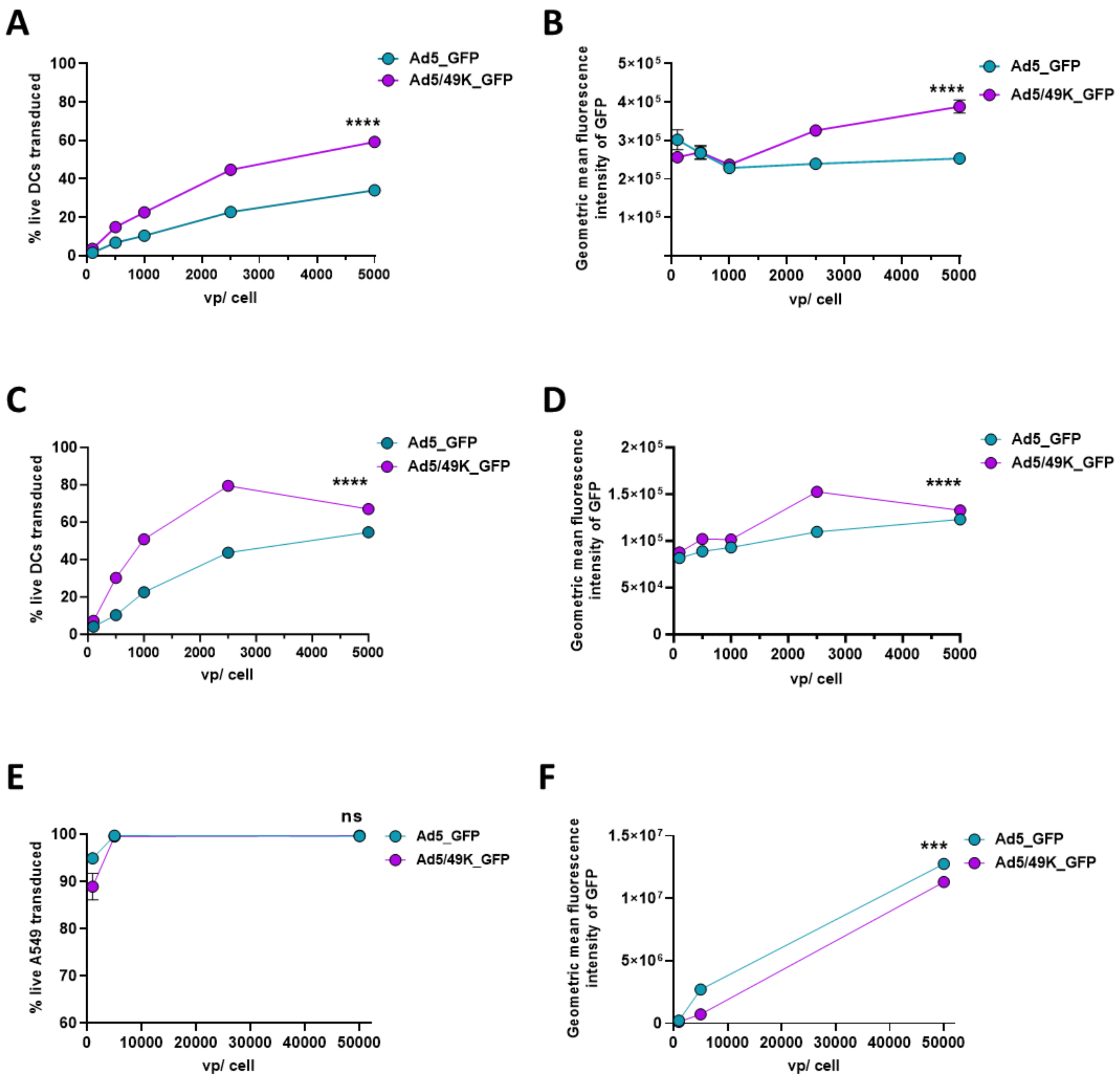

Figure S2 – Area-under-curve analyses of dendritic cell transduction

A-F) Dose response data used to calculate area-under-curve (AUC) of transduction assay data presented in corresponding ***Figure 2A-F***. AUC mean with standard error of the mean (SEM) compared by unpaired T test. Mean and SEM displayed. **** p<0.0001, *** p<0.001, ns = no significant difference.

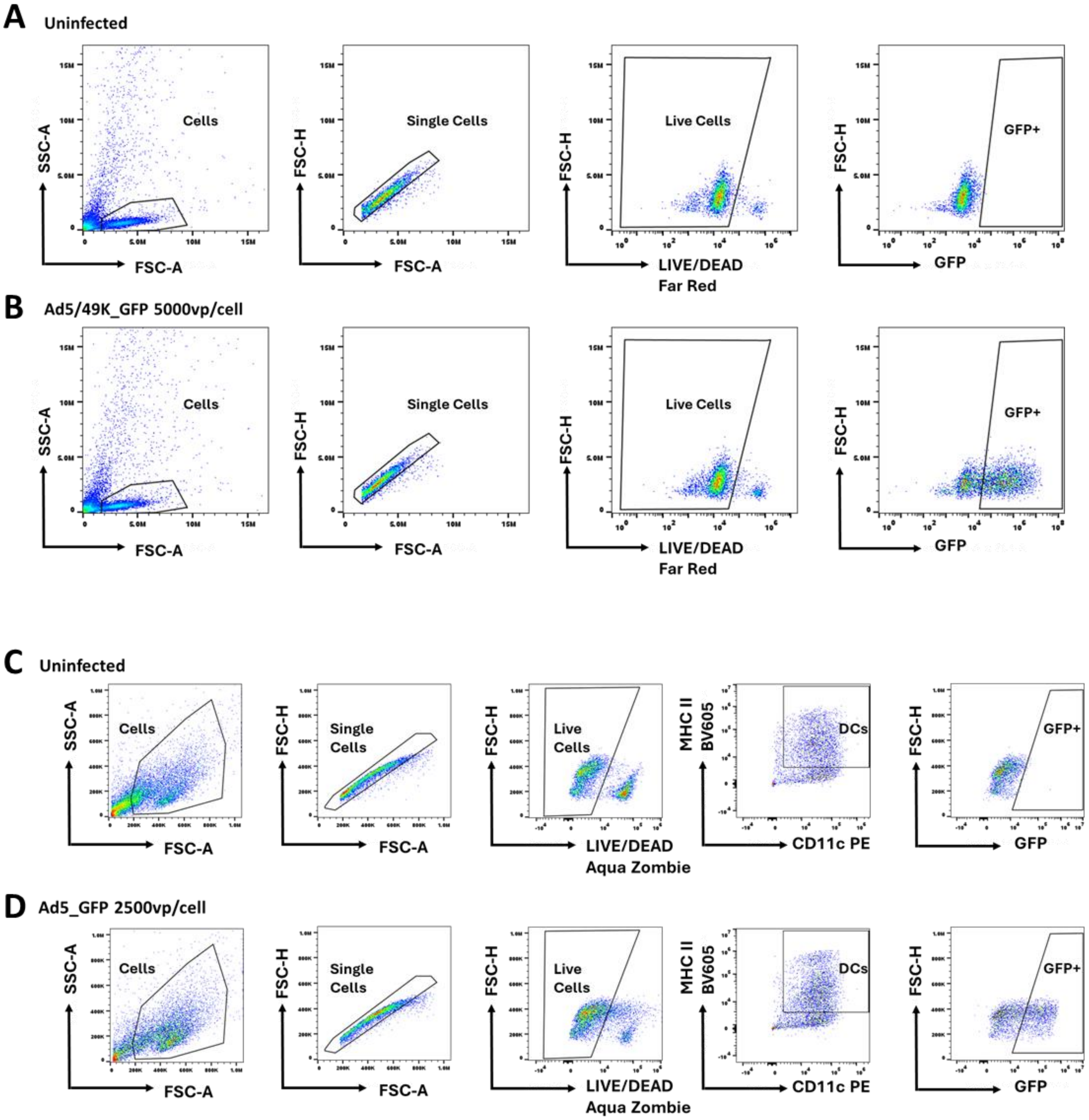

Figure S3 – Human and murine dendritic cell gating strategy for *in vitro* virus transduction assays using GFP-expressing vectors.

Representative gating strategy using Far Red dead cell staining and GFP $^{+}$ gating for **A)** Uninfected monocyte-derived human DCs and **B)** Monocyte-derived human DCs infected with Ad5/49K_GFP at 5,000vp/cell. Representative gating strategy using Aqua Zombie dead cell staining and GFP^{+} gating for **C)** Uninfected bone marrow-derived murine DCs and **D)** Bone-marrow-derived murine DCs infected with Ad5_GFP at 2,500vp/cell.

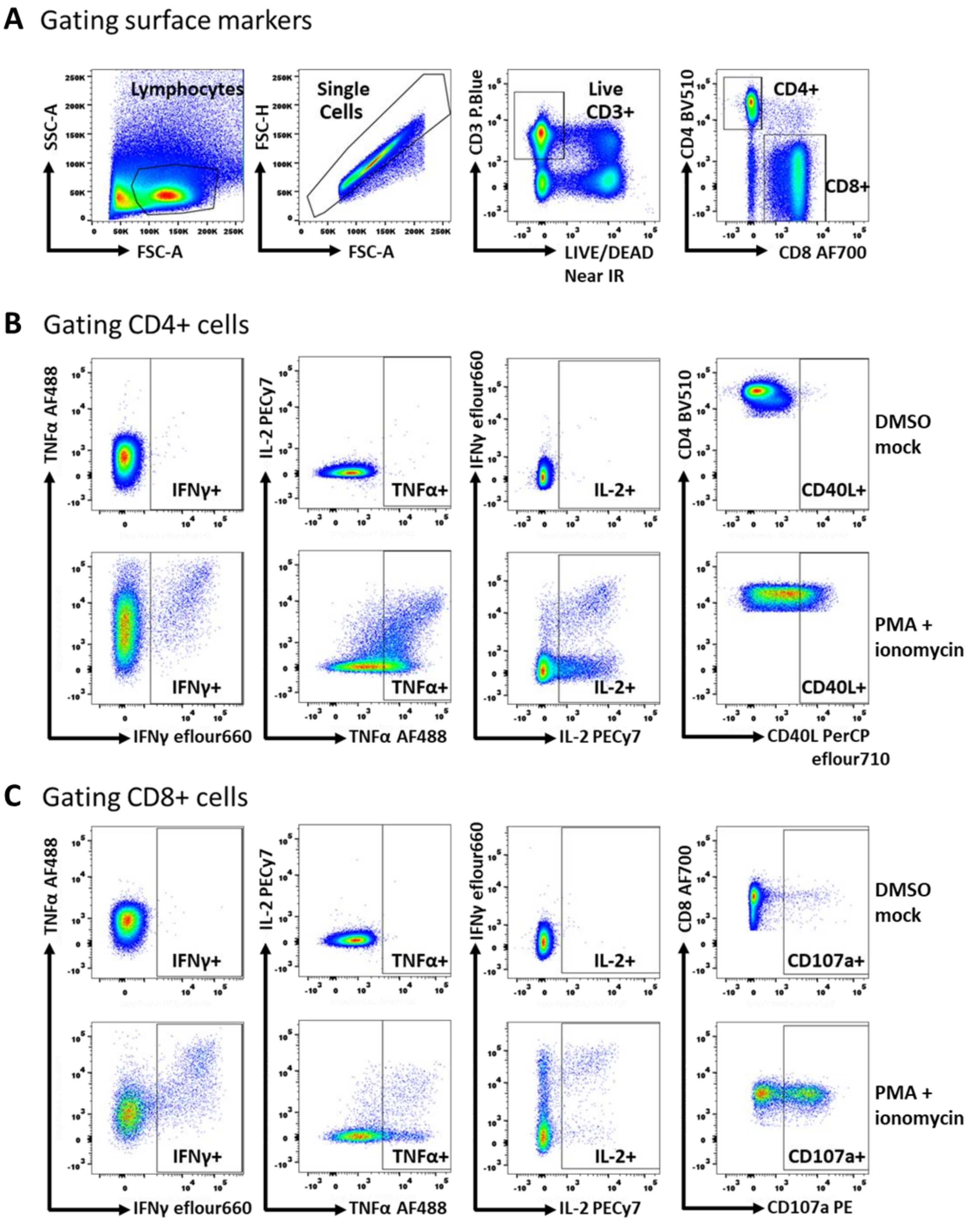

Figure S4 – Gating strategy for flow cytometry with intracellular cytokine staining for vectored vaccines against GFP.

Representative gating strategy for identification of **A)** live $CD4^+$ and $CD8^+$ T cells, with downstream cytokine/CD40L/CD107a responses shown for **B)** $CD4^+$ and **C)** $CD8^+$ populations stimulated with DMSO (-ve control) and PMA+ionomycin (+ve control).

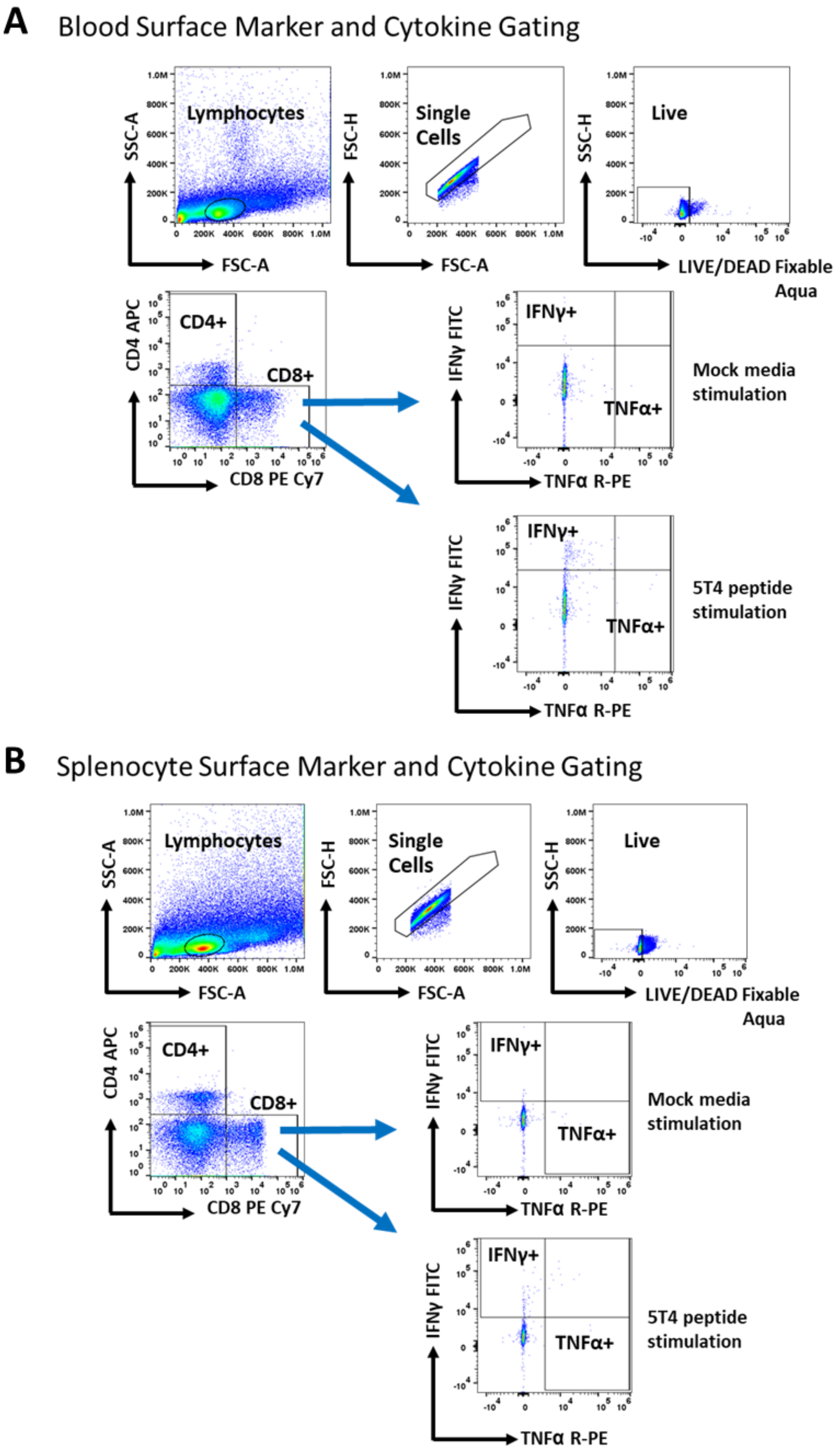

Figure S5 – Gating strategy for flow cytometry with intracellular cytokine staining for vectored vaccines against 5T4.

Representative gating strategy for **A)** PBMC and **B)** splenocyte flow cytometry identification of $CD8^{+}$ T cells with intracellular cytokine staining for media mock (-ve control) and 5T4 peptide stimulated cells.

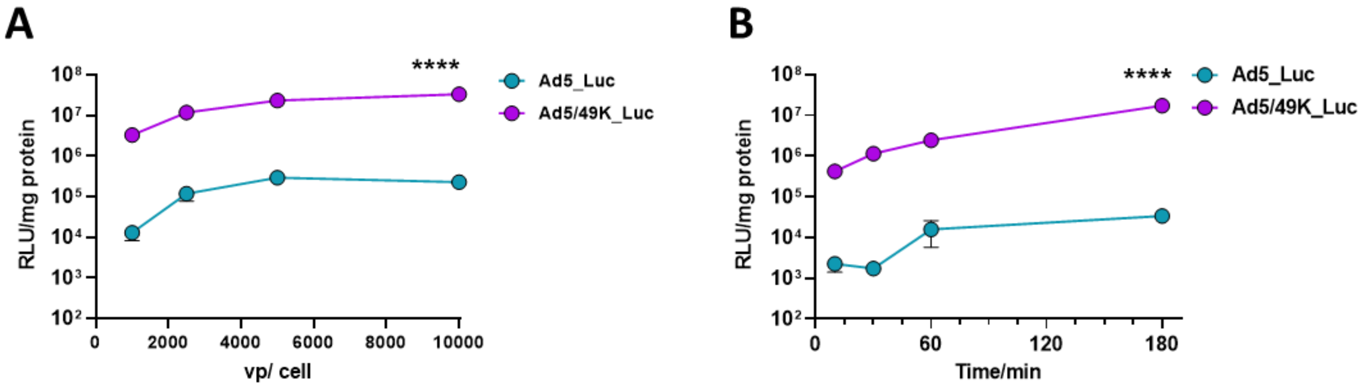

Figure S6 – Area-under-curve analyses of primary human vascular smooth muscle cell transduction.

A-B) Dose response data used to calculate area-under-curve (AUC) of transduction assay data presented in corresponding ***Figure 6A-B***. AUC mean with standard error of the mean (SEM) compared by unpaired T test. Mean and SEM displayed. **** $p<0.0001$.
